# Supplementary material for: Copy number aberrations drive kinase rewiring, leading to genetic vulnerabilities in cancer
Source: Cell Rep. 2021 May 18;35(7):109155. doi: 10.1016/j.celrep.2021.109155 (PMC8149807; doi:10.1016/j.celrep.2021.109155)
Supplement: Document S1. Figures S1–S7 and Table S1 [file mmc1.pdf]

Cell Reports, Volume *35*

Supplemental information

Copy number aberrations drive kinase rewiring, leading to genetic vulnerabilities in cancer

Danish Memon, Michael B. Gill, Evangelia K. Papachristou, David Ochoa, Clive S. D'Santos, Martin L. Miller, and Pedro Beltrao

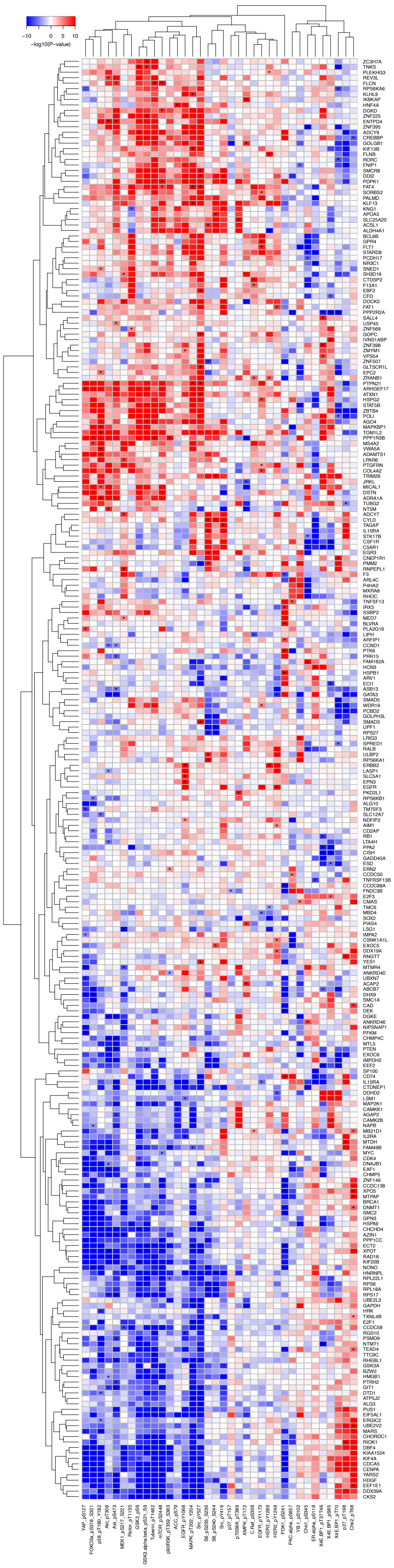

Figure S1. Association between the expression of the predicted driver genes and the activities of the RPPA phosphosites in the TCGA cohort. Significant associations identified from the CNV-phosphosite pipeline are indicated with '*'. Related to Figures 1 and 2.

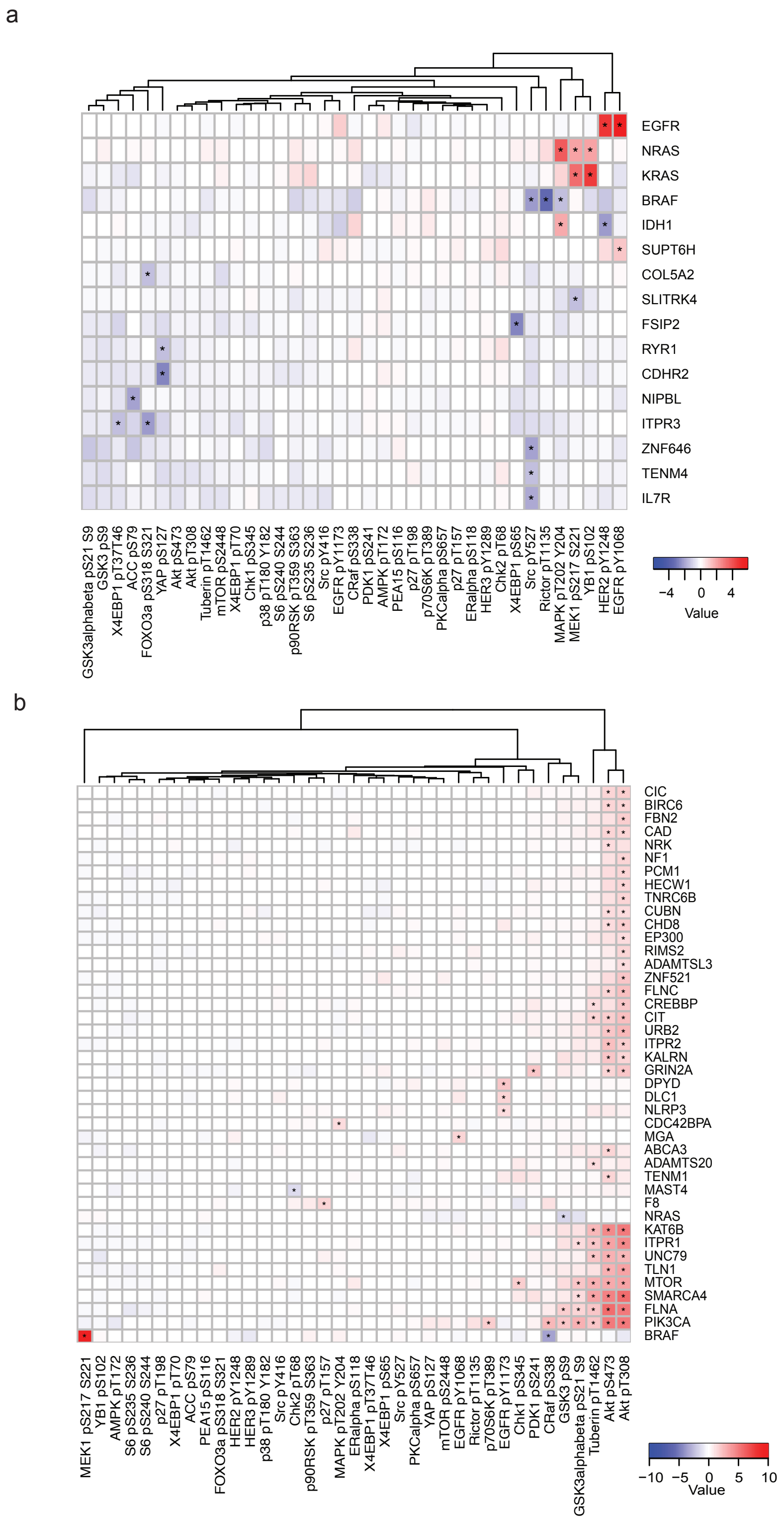

Figure S2. Association between recurrent missense mutations in genes and phosphosite activities in the TCGA cohort and CCLE cohort. (a) The TCGA analysis was restricted to genes with missense mutations in more than 50 out of 4,150 TCGA tumour samples. Only genes with significant association with atleast one phosphosite are shown. * indicates significant associations (FDR < 5%). (b) The CCLE analysis was restricted to genes with missense mutations in more than 50 out of 905 cancer cell lines. Only genes with significant association with atleast one phosphosite are shown. * indicates significant associations (FDR < 5%). Related to Figure 2.

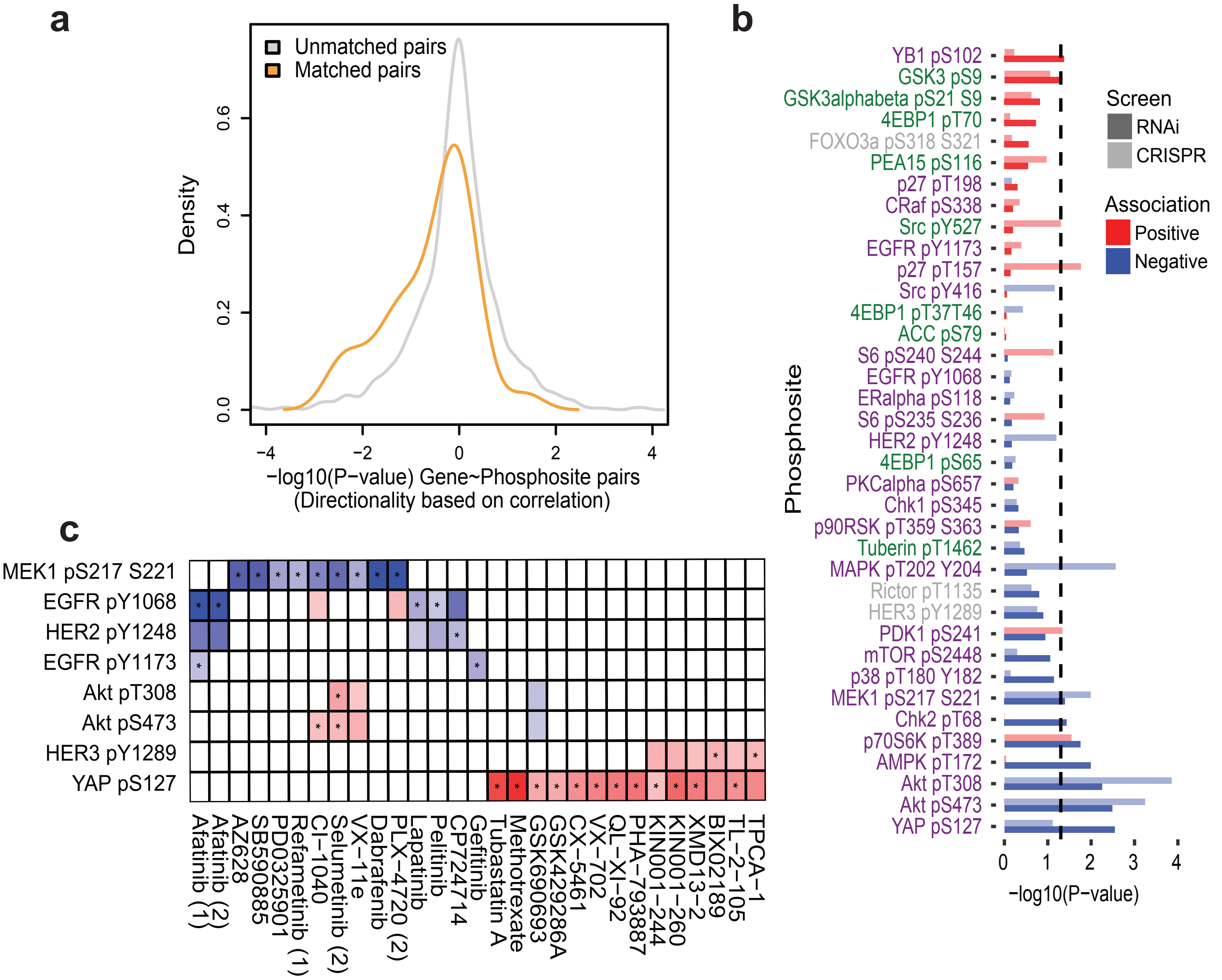

Figure S3. Evidence of kinase addiction in MCLP cancer cell lines. (a) Distribution for the significance of association (-log10(p-value)) between phosphosite activity and gene essentiality (RNAi). (b) significance of association (-log10(p-value)) between phosphosite activity and gene essentiality for each phosphosite using both RNAi and CRISPR Screen. (c) Association between phosphosite activity and drug sensitivity (1% FDR). Positive and negative associations and also supported by RNAi~Drug response are indicated in red and blue respectively. * indicates associations also supported by CRISPR~Drug association. Related to Figure 3.

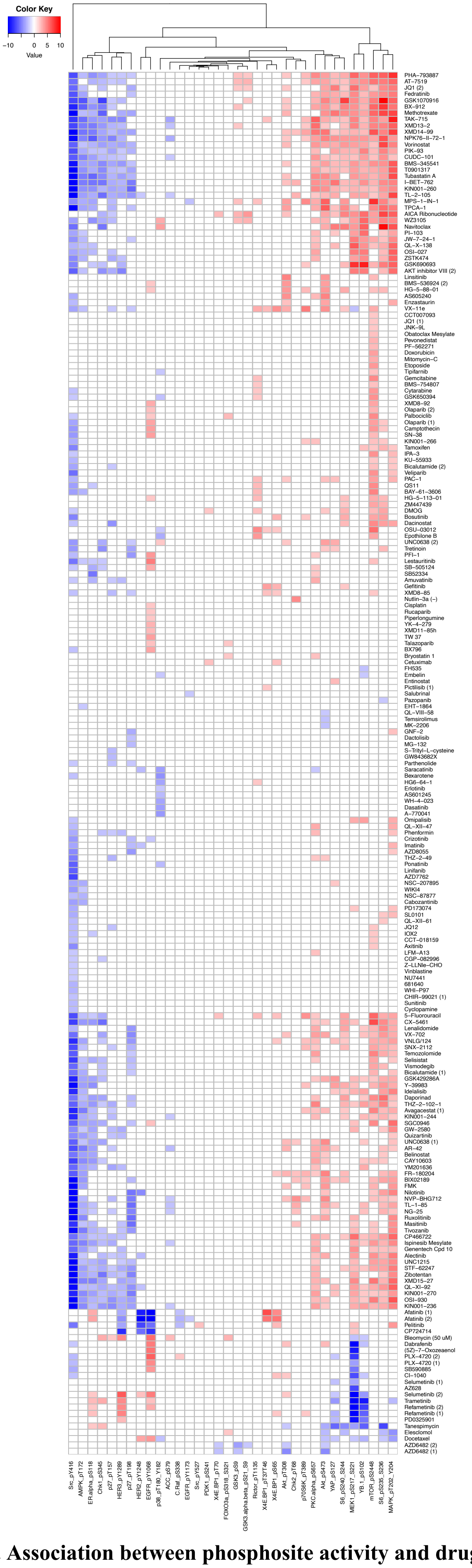

Figure S4. Association between phosphosite activity and drug sensitivity in cancer cell lines. Positive and negative associations that were found to be significant are indicated in red and blue respectively. Related to Figure 3.

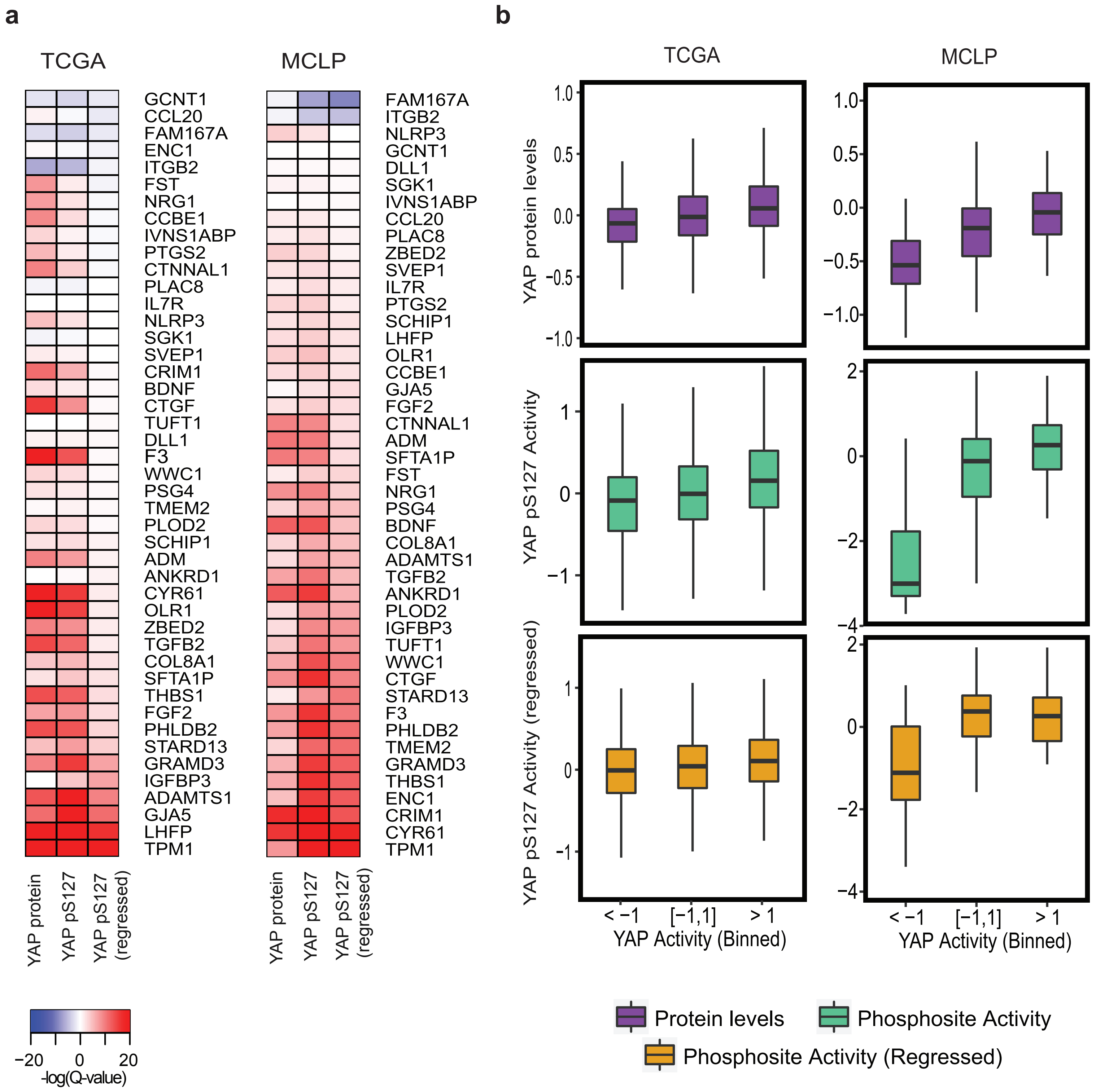

Figure S5. Relationship between YAP protein and YAP pS127 activity and downstream targets of YAP co-factor in TCGA and MCLP Dataset. (a) Correlation between YAP targets and YAP protein and YAP pS127 activity. (b) Difference in YAP protein, YAP and phosphosite levels (before and after regressing out protein changes), binned by levels of YAP co-fac-tor activity. Related to Figure 5.

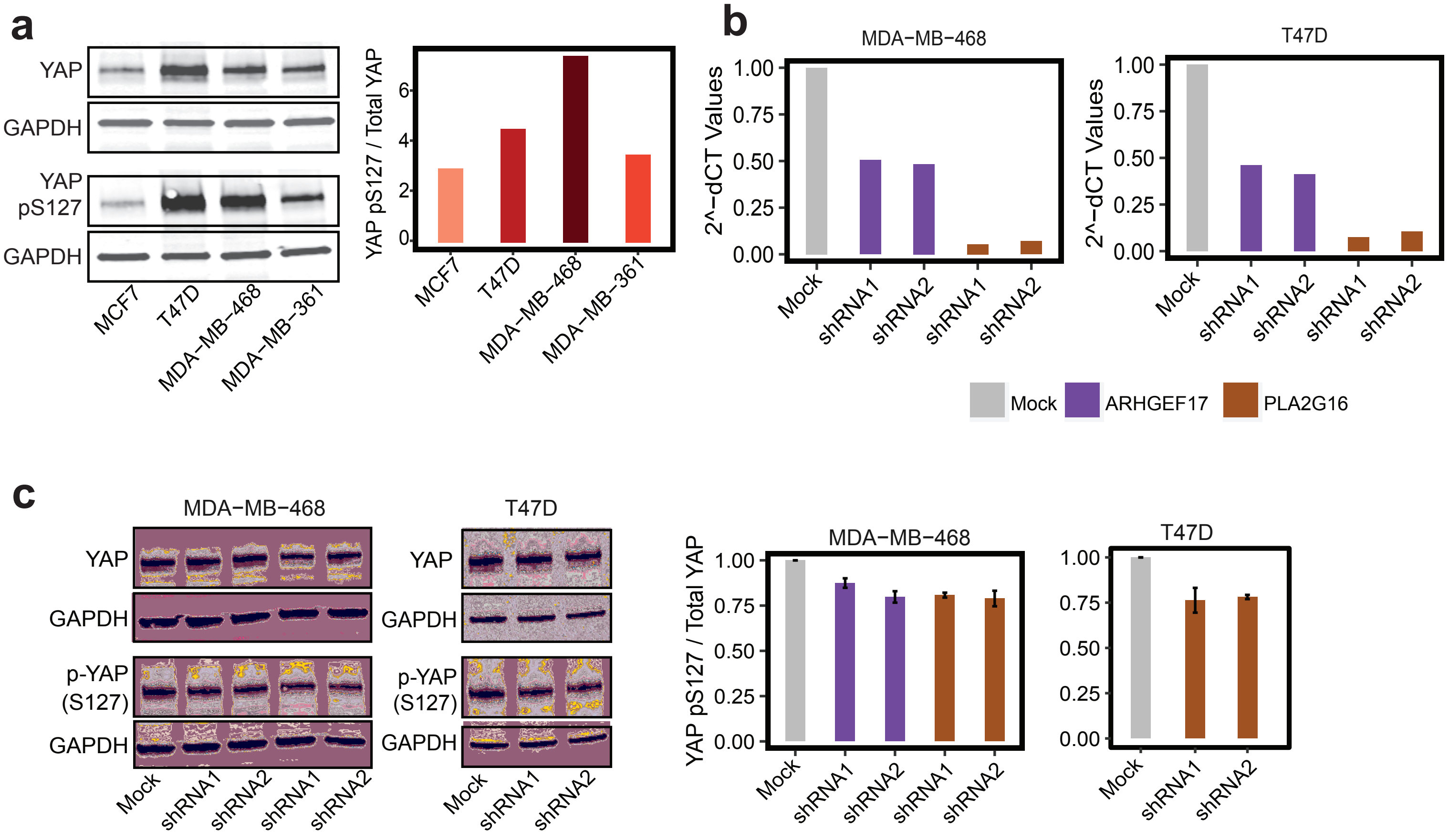

Figure S6 - Knockdown of ARHGEF17 and PLA2G16 modulates the levels of YAP pS127. (a) Western blot analysis of YAP protein and YAP pS127 levels and quantification of relative levels of YAP pS27 in MCF7, T47D MDA-MB-468 and MDA-MB-361 cells. (b) qRT-PCR of ARHGEF17 and PLA2G16 expression levels on shRNA mediated knockdown of ARHGEF17 and PLA2G16 in T47D and MDA-MB-468 cells. (c) Western blot analysis of YAP protein and YAP pS127 levels and quantification of relative YAP pS127 levels on knockdown of ARHGEF17 and PLA2G16 in T47D and MDA-MB-468 cells. Related to Figure 5.

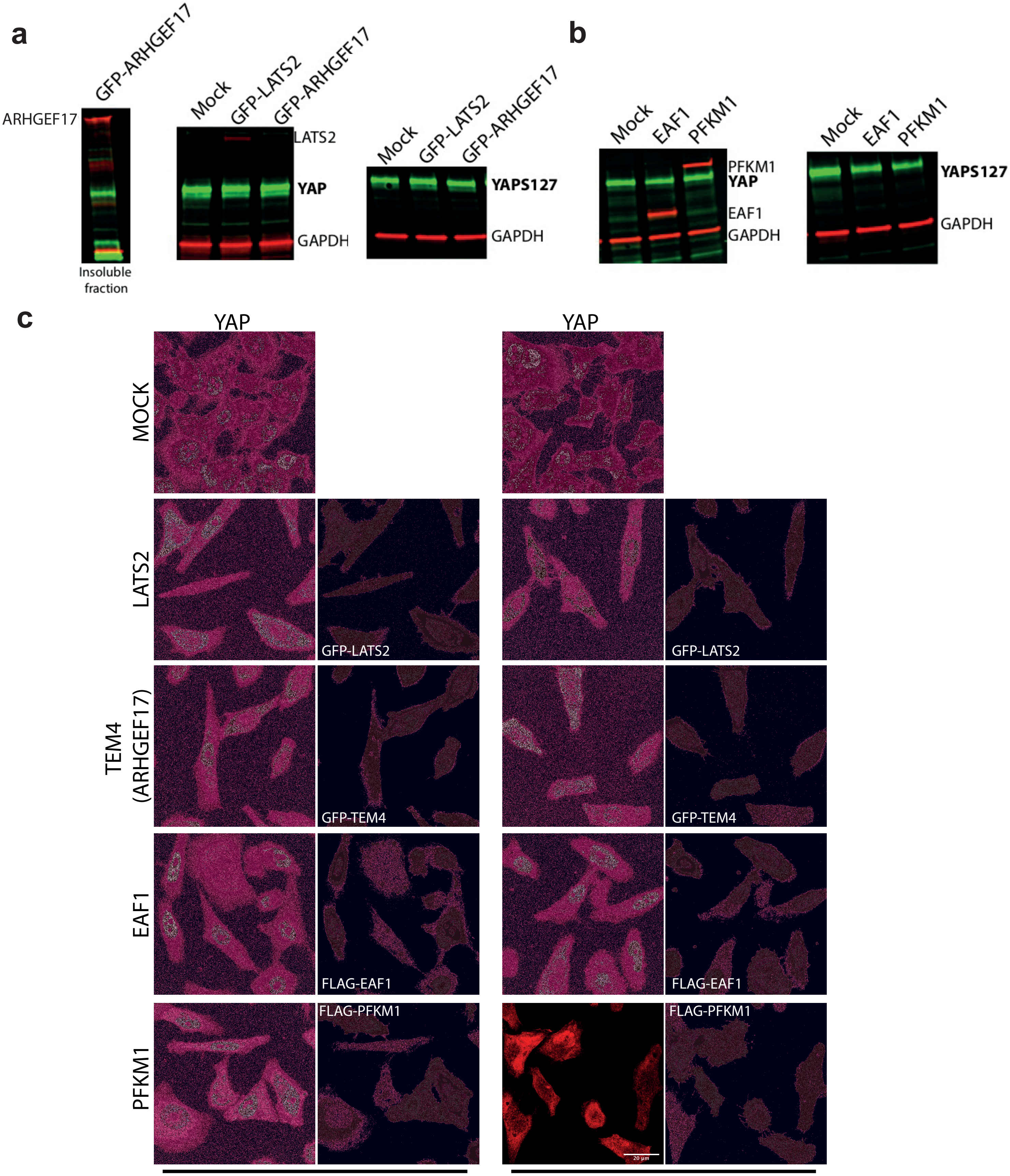

Figure S7 - Over-expression of predicted regulators of hippo-signalling modulates the levels of YAP pS127 and regulates YAP cellular localization. (a) Western blot analysis of YAP protein and YAP pS127 levels on over-expression of ARHGEF17 and LATS2 (positive control). (b) Western blot analysis of YAP protein and YAP pS127 levels on over-expression of EAF1 and PFKM1. (c) Immunofluorescence of YAP protein and YAP pS127 in HeLa cells overexpressing MOCK (control), GFP-LATS2 (positive control), GFP-ARHGEF17, GFP-EAF1 and GFP-PFKM1. Related to Figure 5.

Oligonucleotide	Source
ARHGEF17_shRNA1 (TGCTGTTGACAGTGAGCGCCAGGAGGTTATTCAGAGCATATAGTGAAGCCACAGATGTATATGCTCTGAATAACCTCCTGATGCCTACTGCCTCGGA)	Sigma
ARHGEF17_shRNA2 (TGCTGTTGACAGTGAGCGACATCACCAAGATGGTATCTGATAGTGAAGCCACAGATGTATCAGATACCATCTTGGTGATGGTGCCTACTGCCTCGGA)	Sigma
PLA2G16_shRNA1 (TGCTGTTGACAGTGAGCGCTGGAGTCATGTTCTCAAGAAATAGTGAAGCCACAGATGTATTTCTTGAGAACATGACTCCAATGCCTACTGCCTCGGA)	Sigma
PLA2G16_shRNA2 (TGCTGTTGACAGTGAGCGCGAACTGCGAGCACTTTGTGAATAGTGAAGCCACAGATGTATTCACAAAGTGCTCGCAGTTCTTGCCTACTGCCTCGGA)	Sigma
Primer pairs for PCR:<br>PLA2G16: primer pair 1 **(**AGGCCATCGTGAAGAAGGAA & CAAAGTGCTCGCAGTTCTCA)	Sigma
Primer pairs for PLA2G16; PCR:primer pair 2 **(**AGGAGGTGCTCTACAAGCTG & CTCCATAGCGCAGCTCATTC).	Sigma
Primer pairs for PCR:<br>ARHGEF17**:** primer pair 1 **(**GAGAAGTTGAGCCCATGCTG & GTAGGGCCCTTTCAGACTGT),	Sigma
Primer pairs for PCR:<br>ARHGEF17**:** primer pair 2, primer pair 2 **(**GCGGAAGTCCCTGTCAAATC & ACCCTCAGCTCTGAAAGGTC)**.**	Sigma
36B4/RPLP0: primer pair (GTGTTCGACAATGGCAGCAT & GACACCCTCCAGGAAGCGA)	Sigma

Table S1 - Oligonucleotide information. Related to STAR Methods.
