# Supplementary material for: Copy number aberrations drive kinase rewiring, leading to genetic vulnerabilities in cancer
Source: Cell Rep. 2021 May 18;35(7):109155. doi: 10.1016/j.celrep.2021.109155 (PMC8149807; doi:10.1016/j.celrep.2021.109155)
Supplement: Document S2. Article plus supplemental information [file mmc4.pdf]

Cell Reports

Article

Copy number aberrations drive kinase rewiring, leading to genetic vulnerabilities in cancer

Graphical abstract

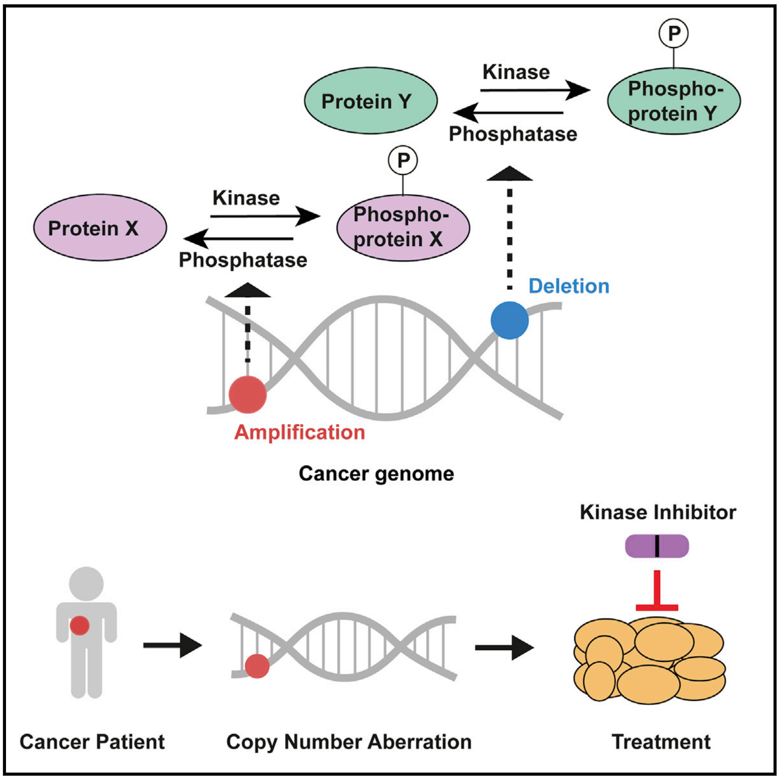

Authors

Danish Memon, Michael B. Gill, Evangelia K. Papachristou, David Ochoa, Clive S. D'Santos, Martin L. Miller, Pedro Beltrao

Correspondence

martin.miller@cruk.cam.ac.uk (M.L.M.), pbeltrao@ebi.ac.uk (P.B.)

In brief

Memon et al. show that copy number alterations can be used as molecular fingerprints to predict the activity of signaling pathways in cancer. Kinase dependencies of cancer cells can be exploited for targeted anticancer therapy.

Highlights

- Alterations in copy number and in signaling pathways are robustly associated in cancer
- Differences in essentiality of phosphoproteins show evidence of kinase addiction
- Copy number markers of dysregulated signaling suggest treatment stratification options

Memon et al., 2021, Cell Reports *35*, 109155
May 18, 2021 © 2021 The Authors.
https://doi.org/10.1016/j.celrep.2021.109155

Cell Reports

CellPress
OPEN ACCESS

Article

Copy number aberrations drive kinase rewiring, leading to genetic vulnerabilities in cancer

Danish Memon,[1,2,3] Michael B. Gill,[2,3] Evangelia K. Papachristou,[2] David Ochoa,[1] Clive S. D'Santos,[2] Martin L. Miller,[2,*] and Pedro Beltrao[1,4,*]
[1]European Molecular Biology Laboratory (EMBL), European Bioinformatics Institute, Wellcome Genome Campus, Hinxton, Cambridge CB10 1SD, UK
[2]Cancer Research UK Cambridge Institute, Li Ka Shing Centre, University of Cambridge, Robinson Way, Cambridge CB2 0RE, UK
[3]These authors contributed equally
[4]Lead contact
*Correspondence: martin.miller@cruk.cam.ac.uk (M.L.M.), pbeltrao@ebi.ac.uk (P.B.)
https://doi.org/10.1016/j.celrep.2021.109155

SUMMARY

Somatic DNA copy number variations (CNVs) are prevalent in cancer and can drive cancer progression, albeit with often uncharacterized roles in altering cell signaling states. Here, we integrate genomic and proteomic data for 5,598 tumor samples to identify CNVs leading to aberrant signal transduction. The resulting associations recapitulate known kinase-substrate relationships, and further network analysis prioritizes likely causal genes. Of the 303 significant associations we identify from the pan-tumor analysis, 43% are replicated in cancer cell lines, including 44 robust gene-phosphosite associations identified across multiple tumor types. Several predicted regulators of hippo signaling are experimentally validated. Using RNAi, CRISPR, and drug screening data, we find evidence of kinase addiction in cancer cell lines, identifying inhibitors for targeting of kinase-dependent cell lines. We propose copy number status of genes as a useful predictor of differential impact of kinase inhibition, a strategy that may be of use in the future for anticancer therapies.

INTRODUCTION

Kinases are druggable proteins that are key targets for cancer treatment, because they are highly prone to acquiring somatic mutations and acting as oncogenes (Gross et al., 2015). Several studies have proposed that cancer cells can become dependent on or addicted to changes in kinase signaling (Sawyers, 2004). However, the challenge remains to identify the genomic context in which specific kinase inhibitors are more likely to be effective. Cancer genomes are characterized by a large number and variety of mutations, including somatic point mutations and copy number aberrations. These mutations may have many direct and indirect effects that are likely to rewire signaling pathways, giving cancer cells a growth advantage. Several studies have reported evidence of somatic mutations that affect kinase activity (Miller et al., 2015; Olow et al., 2016; Reimand and Bader, 2013). However, the impact of copy number aberrations on kinase signaling activity are often unknown, besides some well-characterized cases of copy number variation (CNV) in signaling genes such as PTEN (Chalhoub and Baker, 2009) and ERBB2, which encodes for the HER2 protein (Moasser, 2007). Several tumor types, including breast (Curtis et al., 2012) and ovarian cancer (Macintyre et al., 2018), show large-scale genomic aberrations and have been known to contribute to cancer development and progression. Identifying downstream effects of copy number changes is complicated, because they encompass large segments of genomes with many genes; therefore, it is difficult to identify the likely causal gene within the CNV region.

Large-scale phosphorylation measurements of tumor samples have relied primarily on reverse-phase protein arrays (RPPAs) that consists of (phospho)protein antibody microarrays used to estimate protein abundances in a high-throughput manner. The TCGA (The Cancer Genome Atlas) RPPA platform, TCPA (The Cancer Proteome Atlas), currently has estimates for around 150 proteins and 50 phosphoproteins for nearly 10,000 tumor samples (33 tumor types) (Li et al., 2013). The phosphoproteins measured include extensively annotated functional sites on kinases and transcription factors belonging to key signaling pathways implicated in cancers, including Akt signaling, EGFR signaling, the RAS-RAF pathway, and hippo-signaling pathways. These sites can be used as proxies for kinase signaling in cancer-related pathways.

From the integration of large-scale pan-cancer genomic, transcriptomic, and RPPA phosphoproteomic datasets, we identified genes likely to drive changes in kinase signaling. These associations were found to be enriched in known kinase-substrate relationships and were then systematically filtered to select reliable associations that also replicated by analyzing cancer cell line data. Several predicted regulators of hippo signaling (YAP, ARHGEF17, EAF1, and PFKM) were experimentally tested, and a top-ranked predicted regulator (ARHGEF17) of hippo signaling (YAP) was further studied through (phospho)proteomic analysis. In parallel, we found evidence of kinase addiction in cancer cell

This is an open access article under the CC BY license (http://creativecommons.org/licenses/by/4.0/).

lines and identified inhibitors for targeting of kinase-dependent cell lines. This work suggests that copy number status of genes may be indicative of kinase activity differences and predictive of sensitivity to kinase inhibition. In the long term, this strategy can be used to stratify patients for targeted therapy based on the copy number status of regulators.

RESULTS

Copy number alterations associated with phosphorylation changes in tumors

We have developed a computational method to identify genes driving changes in phosphorylation signaling in patient-derived cancer samples (Figure 1A). A genetic association model was built to predict phosphorylation changes in tumor samples, obtained from RPPA data (TCPA), using CNVs as predictors of signaling events. The changes in phosphorylation, as measured in RPPA, could result from either changes in total protein or changes in the stoichiometry of phosphorylation. To focus on changes in stoichiometry, we restricted our analysis to 37 phosphosites that had matched protein abundances in the RPPA datasets for normalization purposes (STAR Methods). CNVs and gene expression levels were obtained for 15,524 protein-coding genes after excluding genes whose CNVs have been predicted to be post-transcriptionally attenuated (Gonçalves et al., 2017). We then associated CNVs with phosphosite levels of 37 phosphosites across 5,598 tumor samples from 17 tumor types, taking into account total protein abundance and tumor type as covariates (STAR Methods). Because copy number aberrations cover large chromosomal regions, phosphosite changes will show significant CNV associations with many genes in each coamplification region, many of which are likely spurious. As an example, the genes found to be associated with changes in phosphorylation of Akt1 are shown in Figure 1B. On average, each phosphosite was predicted to be associated with 419 genes (10% false discovery rate [FDR], effect size > 0.05).

Before filtering for spurious associations because of coamplification, we tested whether the CNV-phosphosite associations can recapitulate prior knowledge on kinase-substrate relationships. For this, we analyzed 118 known kinase-substrate relationships curated in the PhosphositePlus (Hornbeck et al., 2012) database involving the 37 phosphosites analyzed here. Overall, we observe that known kinase-phosphosite pairs tend to have significant association more often than random expectation ($p < 0.01$) using both CNV and expression levels (Figure 1C). For instance the phosphorylation of YAP pS127, after regression for YAP total protein levels, showed a significant correlation with both expression and copy number changes of LATS2 kinase (Meng et al., 2016). Similarly, expression of PRKD1 (Scheid et al., 2002), PDPK1 (Sato et al., 2002), and MAP3K5 (Gu et al., 2009) kinases are positively correlated with Akt T308, Akt pS473, and p38 pT180,Y182 phosphosites, respectively ($p < 0.01$; Figure 1D).

Given that copy number alterations affect multiple genes within altered regions, many previously defined associations are likely to be spurious passenger associations. We then sought to identify the most likely causal gene controlling the downstream kinase signaling change for each region. First, we selected CNV-associated genes whose expression was also associated with the phosphosite activities (FDR < 5%). We then selected the top-ranked associated genes within each chromosome region based on a combined rank from 3 measurements: (1) the CNV, (2) the gene expression association effect size, and (3) the gene-phosphoprotein distance in a functional interaction network (STAR Methods). We exemplify in Figure 1B the genome-wide gene associations found as putative regulators of AKT1 pT308 phosphorylation levels and the selected causal genes identified within each segment. For this site, we recover the well-known PTEN regulator (Lin et al., 2013), along with other candidate regulators. Using this approach, an average of 8 causal genes per phosphosite were obtained, and each causal gene was a representative of a genomic segment. All phosphosites except for PEA15 pS116 had at least one gene as a predicted regulator, and 12 phosphosites—including Chk1 pS345, Chk2 pT68, Akt pT308, Akt pS473, mitogen-activated protein kinase (MAPK) pT202,Y204, and YAP pS127—were predicted to be regulated by more than 10 causal genes.

We obtained 303 associations between causal genes and regulated phosphosites in the pan-tumor dataset (Figure S1; Table S1). All 303 associations remain significant in the phosphosite-expression and phosphosite-CNV linear model, even after accounting for additional covariates (age, gender, and race). These associations consisted of 264 causal genes, among which 11% (29/264) had more than one association. These genes showed significant enrichment for genes involved in the cell-cycle process, TOR signaling, regulation of protein kinase activity, and positive regulation of response to DNA damage stimulus (Fisher adjusted $p < 0.01$; Figure 1E). The causal genes were also enriched for kinases (19/264; Fisher adjusted $p < 0.01$), including FLT1, PTK6, and YES1, and phosphatases (11/264; Fisher adjusted $p < 0.01$), including PPA2 and PPP1CC. Consistent with their broad-spectrum activity, phosphatases were found to be associated with multiple phosphosites compared with other gene types (Wilcoxon rank-sum $p < 0.05$). For instance, PPP1R3B was predicted to regulate several phosphosites, including p90RSK, Tuberin, and FOXO3 (Table S1). Similarly, PTEN was predicted to regulate both Akt phosphosites (S473 and T308) and GSK3AB pS21,9. We also found significant enrichment for known cancer driver genes from the COSMIC (Catalogue Of Somatic Mutations In Cancer) database in our predicted causal genes list, such as CDK4, EGFR, PTK6, and CCND1 (29/264; Fisher adjusted $p < 0.01$).

Several recurrent protein missense genetic variants are known to cause changes in kinase signaling (Creixell et al., 2015). From 1,002 genes with missense variants in at least 50 patient pan-cancer samples, we found 24 associations between recurrently mutated genes and phosphosite levels (Figure S2). These include a positive association between EGFR mutation and EGFR pY1068 and HER2 pY1248 levels. Similarly, missense mutations in NRAS gene were positively associated with MAPK pT202,Y204 and mutations in KRAS gene were positively associated with MEK1 pS217,S221. In some cases, a copy number event may cooccur with a missense variant, and it is hard to distinguish whether the downstream signaling changes are associated with the copy number change or the point mutation. Therefore, to make sure each of the 303 associations between

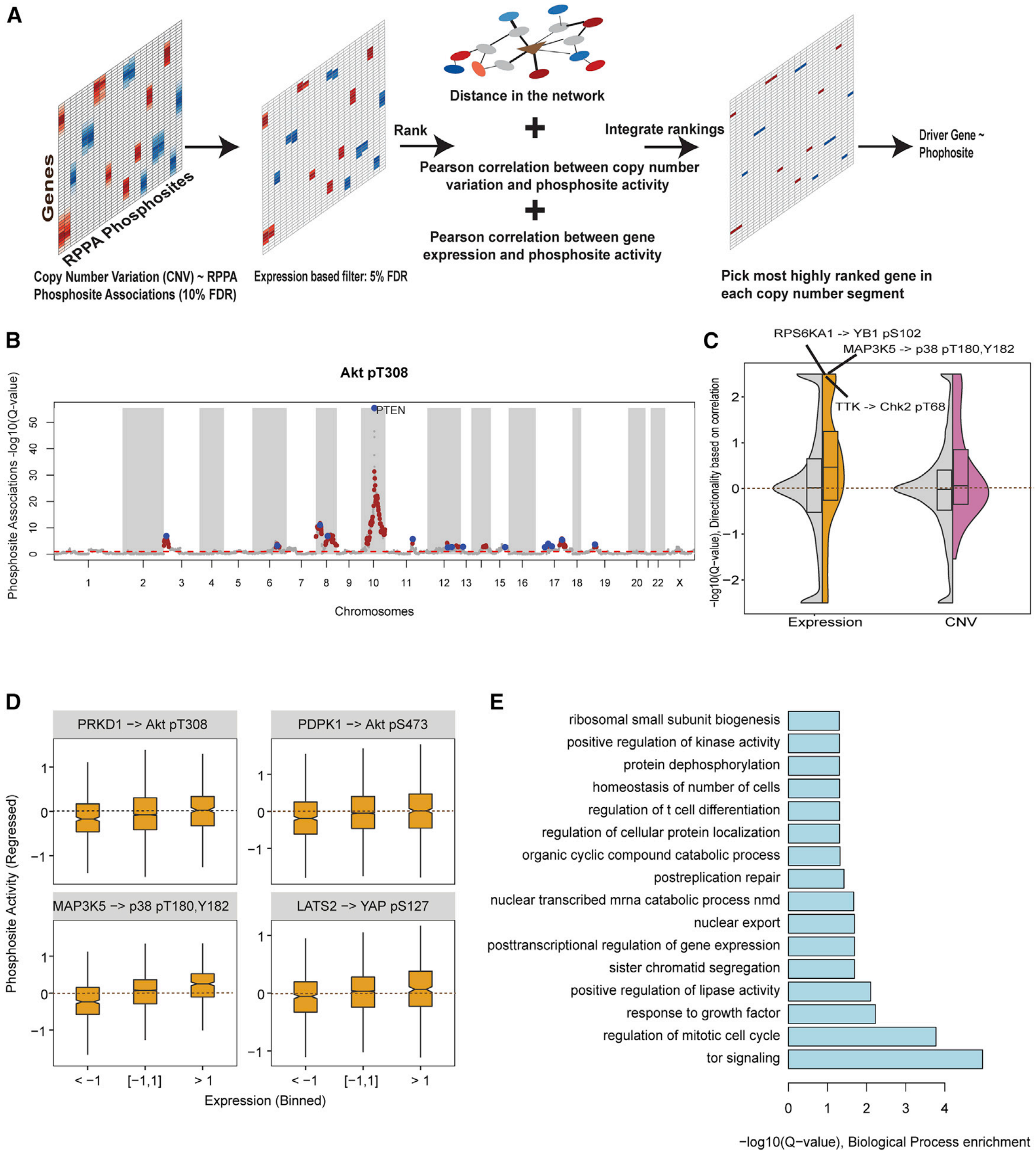

Figure 1. Prediction of regulators of phosphosite levels based on somatic DNA copy number aberrations and gene expression

(A) Key steps in the gene (CNV/expression)-phosphosite phosphorylation association method. Red indicates the strength of positive associations, and blue represents the strength of negative associations. The schematic network represents protein-protein interactions in the STRING (Search Tool for the Retrieval of Interacting Genes/Proteins) database, with nodes as proteins or genes and edges as interactions. Edge thickness is indicative of the strength of interaction in the network. A network-based score derived from the distance between CNV (gene) node and phosphosite (gene) node in the network and the weights of interactions separating these nodes was used to rank the associations.

(B) Manhattan plot showing the genome-wide associations between gene copy number changes and Akt phosphosite levels (Akt pT308). The dashed red line indicates the cutoff for significant copy-number-based associations (10% FDR). Genes highlighted in brown are supported by gene-expression-based associations. Genes highlighted in blue are the top-ranked genes within each genomic region.

(C) Significance of correlation between CNV (in purple) or mRNA (in yellow) levels with phosphosite changes for known kinase-substrate interactions (colored) or any gene-phosphosite pair (in gray). Positive/negative signs indicate directionality of associations.

(D) RPPA phosphosite levels, after regressing away protein changes, binned by mRNA levels of regulators in pan-cancer data from TCGA/TCPA for known regulator-substrate relationships.

(E) Gene Ontology overrepresentation analysis of predicted regulators.

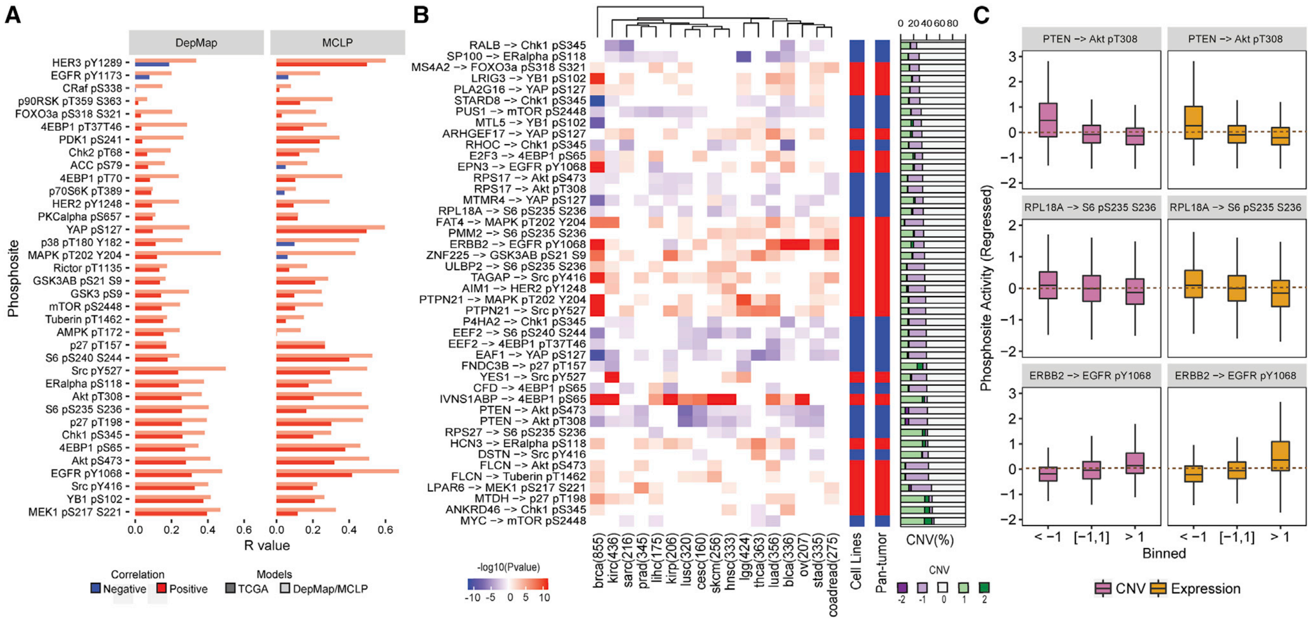

Figure 2. Shortlisted gene-phosphosite pairs replicated in cancer cell line data

(A) Variation explained in phosphosite levels as predicted by a linear model for each phosphosite based on expression of putative causal genes. Linear models derived from RPPA data based on TCGA (dark), DepMap (light), and MCLP (light) were used to predict phosphosite levels on expression data based cell lines and correlated with the measured DepMap and MCLP RPPA measurements.

(B) Shortlist of gene-phosphosite pairs identified in pan-tumor analysis and replicated in both DepMap and MCLP cell lines.

(C) Changes in phosphosite levels (after regressing out protein changes), binned by mRNA levels or copy number levels of predicted regulators for some top-ranked and replicated pairs.

copy number changes and phosphosites is independent of cooccurring point mutations, we iteratively tested the impact of each recurrently mutated gene as a covariate in the CNV-phosphosite linear model. Although in some cases there was a decrease in significance for the CNV-phosphosite associations, in only 6 cases the CNVs were not a significant added predictor. For example, the association between PDPK1 CNV and mTOR pS2448 levels could be explained by missense mutations in IL7R. Similarly, association between ZMYM1 CNV and EGFR pY1068 could be explained by mutations in EGFR.

Replication of predicted regulators of signaling changes in cancer cell lines

To investigate the reliability of the predicted associations from the pan-tumor analysis, we attempted to replicate the significant associations in the cancer cell line cohort from CCLE (Cancer Cell Line Encyclopedia) with publicly available copy number (Barretina et al., 2012), expression (Barretina et al., 2012), and phosphorylation (Zhao et al., 2017) information. We interrogated two independent RPPA datasets from DepMap and MCLP (MD Anderson Cell Lines Project) to test the replicability of these associations in cancer cell lines. A total of 130 of 303 of the predicted causal gene-phosphosite associations were replicated in the larger set of 890 cancer cell lines in DepMap (10% FDR; Table S1). Separately, 66 of 303 predicted causal gene-phosphosite were replicated in the smaller set of 319 cancer cell lines in MCLP (10% FDR; Table S1), using the mRNA levels of the predicted causal genes. Differences in predictions may partially result from differences in sample size and/or differences in heterogeneity of the tumor versus the cell line samples, such as presence of non-tumor cells or changes during *in vitro* culturing. To assess the overall predictive power of levels of individual phosphosites, we trained models of phosphosite levels as a linear combination of expression profiles of the predicted causal genes (STAR Methods). For 8 of 37 phosphosites, at least 20% of the variance in phosphosite levels in DepMap cell lines could be significantly explained by the expression of predicted causal genes in the cell lines. One of these phosphosites, MEK1 pS217,S221, at least 20% of the variance in levels in DepMap cell lines could be predicted using a linear model trained on the TCGA tumor dataset. For 12 of 37 phosphosites, at least 20% of the variance in phosphosite levels in MCLP cell lines could be significantly explained by the expression of predicted causal genes (Figure 2A). For three of these phosphosites, HER3 pY1289, EGFR pY1068, and YAP pS127, at least 20% of the variance in level in the MCLP cell lines could be predicted using the linear model trained on the TCGA tumor dataset (Figure 2A).

A stringent set of 44 causal gene-phosphosite pairs were defined that were replicated in at least 20% of tumor types (4/18) and replicated independently in two cancer cell line datasets (Figure 2B). Among the replicated associations were well-known examples (Figures 2B and 2C) such as loss of PTEN correlated with increase in phosphorylation of Akt pT308 and Akt pS473 (Lin et al., 2013) and EGFR amplification correlated with HER2 pY1248 phosphosite level (Dittmar et al., 2002). In addition to

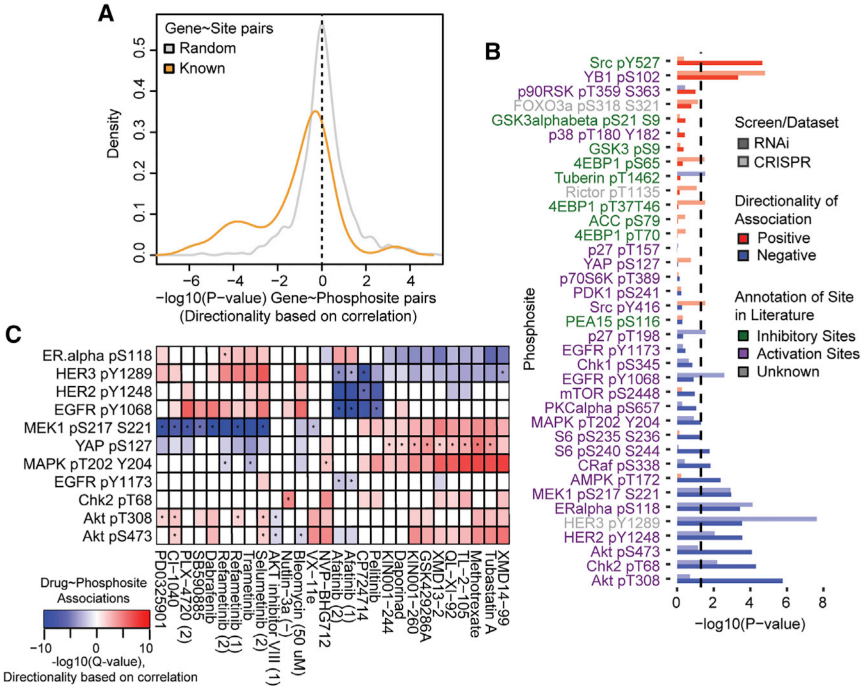

Figure 3. Evidence of kinase addiction in DepMap cancer cell lines

(A) Distribution for the significance of association ($-\log_{10}$(p value)) between phosphosite level and gene essentiality (CRISPR).

(B) Significance of association ($-\log_{10}$(p value)) between phosphosite level and gene essentiality for each phosphosite using both RNAi and CRISPR screen.

(C) Association between phosphosite level and drug sensitivity (1% FDR). Positive and negative associations and supported by RNAi-drug response are indicated in red and blue, respectively.

Asterisk (*) indicates associations also supported by CRISPR-drug association. Only drugs with at least one significant association with a phosphosite and supported by their association with CRISPR and RNAi screen are shown in this figure. All associations are shown in Figure S4.

these known examples, we were able to identify several potential regulators of phosphosite levels that have been previously reported to play a key role in cancer development and/or progression. For instance, the MTDH gene known to be involved in the regulation of several signaling pathways—including Akt, Wnt, and MAPK, leading to tumor metastasis (Emdad et al., 2013)—was predicted to positively regulate the p27 pT198 (cyclin-dependent kinase inhibitor) level.

Evidence of kinase addiction in cancer cells

Having shown that copy number alterations lead to kinase signaling changes, we next sought to find cases in which such signaling differences create potential genetic vulnerabilities. Previous studies have proposed that cancer cells have a tendency to become dependent on the activity of proteins such as kinases (Tyner et al., 2013) and are therefore likely to be sensitive to loss of the kinase gene. We exploited the recently generated RNAi (Tsherniak et al., 2017) and CRISPR (Meyers et al., 2017) knockdown and knockout (KO) data for cancer cell lines in Project Achilles to test the generality of this concept. RPPA phosphosites were classified as activating (25 sites) or inhibitory (9 sites) based on evidence in the literature (Hornbeck et al., 2012; Korkut et al., 2015). Protein activities based on phosphosite levels in DepMap were then correlated with sensitivity to loss of the corresponding gene across 406 cancer cell lines in the CRISPR screen. Increased phosphosite levels in a given cell line showed an overall tendency for increased essentiality of the gene in the respective cell line compared with random pairs (Wilcoxon rank-sum $p < 0.05$; Figure 3A). Activation sites showed an overall tendency for negative correlation, and inhibitory sites showed an overall tendency for positive correlation (Figure 3B). However, the protein activity dependencies were only significant for a fraction of the regulated proteins (Figure 3B). These dependencies varied across genes and were recapitulated for several proteins using RNAi data with matched RPPA phosphoproteomic measurements (Figure 3B). The strongest reproduced activity dependencies were observed for YB1, AKT, Her2, ER alpha, and MEK, with other strong dependencies observed in at least one screen, i.e., CRISPR and RNAi, but not both. We repeated the same analysis of associations between RNAi/CRISPR screens with RPPA data from the MCLP dataset and observed the same trend of phosphosite levels to be correlated with cellular sensitivity to loss of the phosphosite gene, although stronger associations were observed for different phosphosites, such as YAP pS127 (Figure S3). This analysis confirms that on average, cancer cell lines show increased dependency on the activity of a subset of kinases and other proteins and are sensitive to their inhibition.

We next investigated drug response datasets to interrogate the relationship between phosphosite levels and drugs. We correlated phosphosite levels as measured by 37 phospho-Ab with sensitivity profiles of 265 drugs in 746 cancer cell lines from Genomics of Drug Sensitivity in Cancer (GDSC) database (Yang et al., 2013), in which 30 phosphosites showed significant correlation with sensitivity of at least one drug target (FDR < 5%; Figure S4). A substantial fraction of these phosphosites (18/30) was associated with sensitivity to more than 25 drugs. For instance, cancer cell lines with a high MEK1 pS217,S221 level were more sensitive to most RAF/MEK/ERK inhibitors (such as refametinib, SB590885). Similarly, the YAP pS127 level correlated with methotrexate inhibition. We refined these associations to identify 870 cases in which the drug sensitivity was correlated with sensitivity to loss of the kinase/phosphosite gene in either RNAi or CRISPR screen (Figure 3C). In 31 of these cases, there was a concordance in both gene perturbation screens. For instance, cell lines sensitive to EGFR inhibitors (pelitinib and afatinib), as predicted by the high EGFR phosphosite level, were also sensitive to loss of the EGFR gene in both CRISPR and

RNAi datasets. Similarly, cell lines sensitive to ERBB2 inhibitor (CP724714), correlated with the high HER2 pY1248 and HER3 pY1289 phosphosite level, were also sensitive to loss of the ERBB2 gene in both CRISPR and RNAi datasets. Many of these associations between phosphosite level and drug response were replicated using the RPPA data from MCLP (Figure S3).

CNVs as predictors of kinase-related genetic susceptibility

Our results indicate that copy number changes in genes can be indicative of changes in phosphosite levels and that cancer cells can become dependent on activity as measured by these phosphosites. Therefore, copy number changes correlated with kinase activity should also be predictive of kinase susceptibility. Indeed, we found several examples in which the CNVs predictive of phosphosite level differences correlated with the impact of downregulation, knockout, or inhibition of the corresponding protein. For instance, ERBB2 and EPN3 CNVs were predictive of EGFR pS1068 phosphosite levels and correlated with sensitivity to loss of the EGFR gene and sensitivity to EGFR inhibitors, including afatinib and gefitinib (Figures 4A and 4B).

As an additional example, we looked more in detail at the CNV-phosphosite association for YAP pS127, identifying 12 genes as potential regulators, 5 of which were replicated in cancer cell lines (Figure 5A). We ranked the genes based on their association with YAP phosphosite levels in a multiple linear regression model using tumor (TCGA) and the two cell line RPPA datasets (Figure 5B). In the combined model, the CNVs of ARHGEF17, EAF1, PFKM, and PLA2G16 showed the strongest association with YAP pS127 levels (Figure 5B). YAP, a transcription cofactor, is a key component of the hippo-signaling pathway (Gumbiner and Kim, 2014). Phosphorylation of YAP at S127 leads to the retention of YAP in the cytoplasm (Zhao et al., 2007), and further phosphorylation of YAP at S381 and S384 is associated with YAP degradation (Zhao et al., 2010). Therefore, the phosphorylation of S127 should result in a decrease in the transcriptional activity of YAP. However, phosphorylation at S128 can also block the 14-3-3-mediated cytoplasmic retention of S127 phospho-YAP (Moon et al., 2017). To disambiguate the transcriptional activity changes as detected by the S127 RPPA antibody, we studied the gene expression changes of 45 positively regulated targets of YAP (Kim et al., 2015). We observed on average a positive correlation between the changes in expression of these genes and the changes in total YAP, total phosphorylated YAP, and phosphorylated YAP after normalizing for YAP protein levels (Figure S5). This was the case in both patient tumor samples and MCLP cell lines (Figure S5). This would suggest that the RPPA antibody for S127 is positively associated with YAP activity. In line with this, there is a positive correlation between the RPPA YAP S127 levels and the sensitivity of cells to knockdown or knockout of YAP (Figure S5).

We experimentally tested the effect of loss of ARHGEF17 and PLA2G16 genes on YAP pS127 phosphosite levels. We selected cell lines with different baseline levels of YAP phosphorylation and confirmed that YAP pS127 levels were low in MCF7 and MDA-MB-361 cells and high in MDA-MB-468 and T47D cells, consistent with the measurements in the MCLP RPPA phosphoproteomics dataset from the cancer cell line cohort (Figure S6). Effective lentiviral short hairpin RNA (shRNA)-mediated knockdown of both ARHGEF17 and PLA2G16 in MDA-MB-468 and T47D cells was confirmed using qRT-PCR (Figure 5C; Figure S6). Knockdown of ARHGEF17 resulted in a small but significant decrease in the levels of YAP pS127 in both cell lines, which was consistent with the predictions ($p < 0.05$; Figures 5C and 5D). Loss of PLA2G16 also had a reproducible but modest effect on YAP pS127 (Figure S6). Separately, we experimentally tested the overexpression of ARHGEF17, PFKM, and EAF1 on YAP pS127 phosphosite levels in HeLa cells. Overexpression of LATS2 (a positive control) and ARHGEF17 led to an increase in YAP pS127 levels and localization of YAP protein in the cytoplasm, indicative of YAP pS127 phosphorylation (Figure 5E; Figure S7). In contrast, overexpression of PFKM and EAF1 showed a modest decrease in YAP pS127 levels and localization of YAP protein in the nucleus, consistent with the associations based on the linear model (Figure 5E; Figure S7). These results indicate that the identified associations can often be reproduced by perturbation experiments in model cell lines.

To further dissect the regulatory role of ARHGEF17 in hippo signaling, we performed proteomics and phosphoproteomics of MDA-MB-468 and T47D cells treated with shRNA targeting ARHGEF17, along with their matched controls. Differential phosphosite analysis data showed significant changes in 3,182 and 259 phosphosites after ARHGEF17 knockdown (5% FDR) in MDA-MB-468 and T47D cells, respectively (Table S2). These include significant decrease in multiple YAP phosphosites in both cell lines (Figure 5F). We then studied the protein abundance changes and particularly that of components of the hippo-signaling pathway upon ARHGEF17 knockdown (Figure 5G). Differential protein abundance analysis showed significant changes in 1,754 and 308 proteins after ARHGEF17 knockdown (5% FDR) in MDA-MB-468 and T47D cells, respectively (Table S2), which include the expected decrease in ARHGEF17 protein levels in both cell lines (adj. $p < 0.01$). We also observed a significant decrease in YAP protein levels in cell lines that, despite our normalization efforts, may explain most changes in phosphorylation (Figure 5H). Several core components of the hippo pathway, including STK3, STK38, and SAV1, were also found to be significantly dysregulated in MDA-MB-468 cell lines (Figure 5H). We again assessed the changes in YAP cofactor activity, derived from positively regulated targets of YAP, observing a significant decrease in T47D cells ($p < 0.01$) and a more modest decrease in MDA-MB-468 cells (Figure 5I).

Altogether, these analyses suggest that ARHGEF17, EAF1, PFKM, and PLA2G16 copy number changes can be linked with changes in YAP signaling. Across the tumor samples, increased expression of ARHGEF17 is associated with increased YAP activity and increased sensitivity to loss of the YAP gene and interestingly is linked with decreased sensitivity to methotrexate (Figure 5J). This example further illustrates how copy number or expression changes of the regulators identified in this study can be used to gain insights into the genetic vulnerabilities of tumors.

DISCUSSION

We propose here a genetic association approach to identify genes whose expression changes can drive differences in kinase

CellPress
OPEN ACCESS

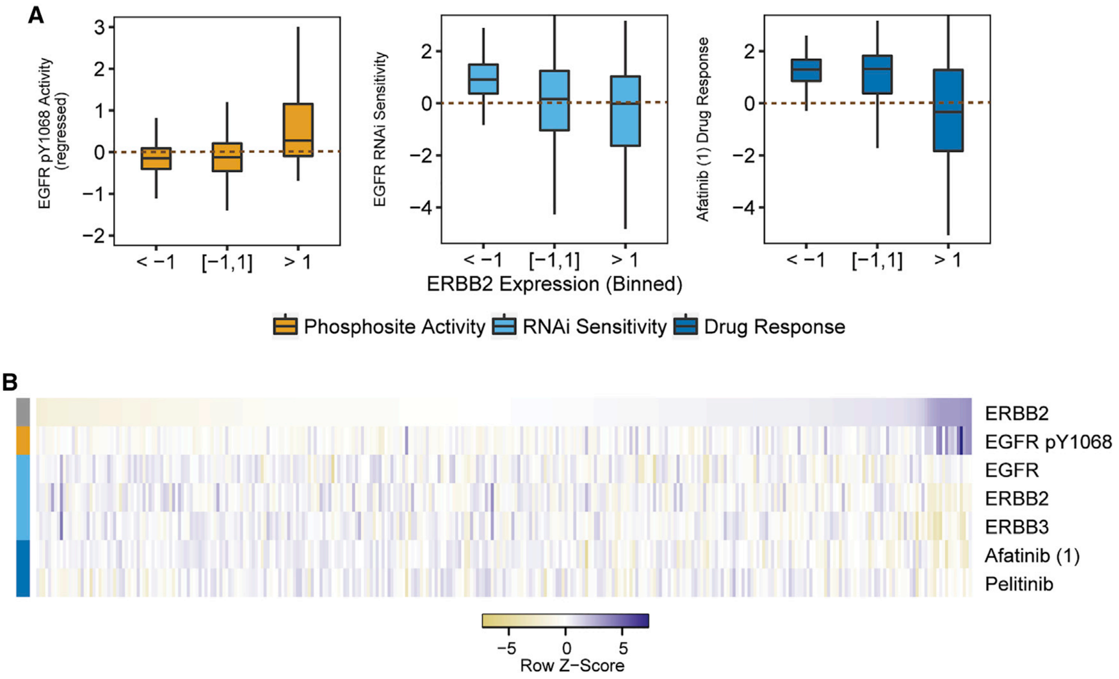

Figure 4. CNVs as predictors for kinase-related genetic susceptibility

(A and B) Levels of EGFR pY1068 activity, RNAi sensitivity to loss of the EGFR (and ERBB2 and ERBB3) gene, and sensitivity to related kinase inhibitors when (A) binned or (B) ordered according to ERBB2 expression levels.

signaling. Such regulators are unlikely to be direct, because they are found through genetic association for differences in steady-state phosphorylation levels. We use copy number variation instead of recurrent missense mutations, given that they form a critical mass of genomic aberrations encountered in multiple tumor types and compared with point mutations, the impact of copy number changes on an individual gene is easier to interpret. For most genes, their copy number profiles tend to be well correlated with gene expression data (Hyman et al., 2002). Although the degree of changes and interpretability makes CNVs an attractive choice for association analysis, these changes typically involve a large number of genes and in some cases entire chromosomal segments. In these scenarios, it is a challenge to identify the key regulator of downstream signaling changes among the cofactor or codeleted block of genes. Here we have defined an approach to prioritize copy number events that are regulators of signaling pathways, showing that the method was able to recover previously known regulatory relationships. However, it is possible that the top-selected gene will not be the driver gene for the associated genomic region, and we provide in the Table S1 the rank of genes associated with a phosphosite for each segment.

We selected for further analysis several putative regulators of YAP phosphorylation, including ARHGEF17 that we followed up through (phospho)proteomics studies. ARHGEF17 is a Rho guanosine triphosphatase (GTPase) with a functional role in the maintenance of actin cytoskeleton organization and focal adhesion (Mitin et al., 2013). In the tumor patient samples, cancer cell lines, and direct knockdown experiments, the levels of ARHGEF17 were positively correlated with the transcriptional activity of YAP. However, the relationship between YAP phosphorylation and protein levels is not easy to disentangle, and we cannot rule out, based on the current set of results, that the ARHGEF17-mediated regulation of YAP is primarily driven by protein-level changes. Moreover, the effects of varying ARHGEF17 levels on phosphorylation of YAP by shRNA-mediated knockdown are low, and the association remains to be corroborated in future studies.

The current study relied on antibody measurement of phosphorylation and protein abundance levels of proteins with reliable antibodies and previous evidence of a role in cancer. This approach has great coverage, allowing us to identify associations from ~5,600 samples and thus providing reliable significant associations among key signaling changes in cancer. However, our analysis is focused on a restricted set of signaling pathways, and inevitably, we are unable to identify regulators of less studied pathways. More recently, mass spectrometry has started to be used to profile tumor samples on a large scale for an increasing number of samples (Edwards et al., 2015). Although the current limited set of samples makes it difficult to perform analysis at the genome-wide association level, as these efforts progress, the approach described here can be applied to those data, potentially providing a higher-resolution description of how different CNVs rewire kinase signaling in cancer.

Cancer cells often become dependent on changes in the genes that led to the cancer development, a phenomenon dubbed oncogene addiction. When a signaling pathway is observed to be hyperactive in a tumor, relative to healthy tissue, it is often assumed that such activation is a driving event and a requirement for cell viability. Therefore, cellular dependency on

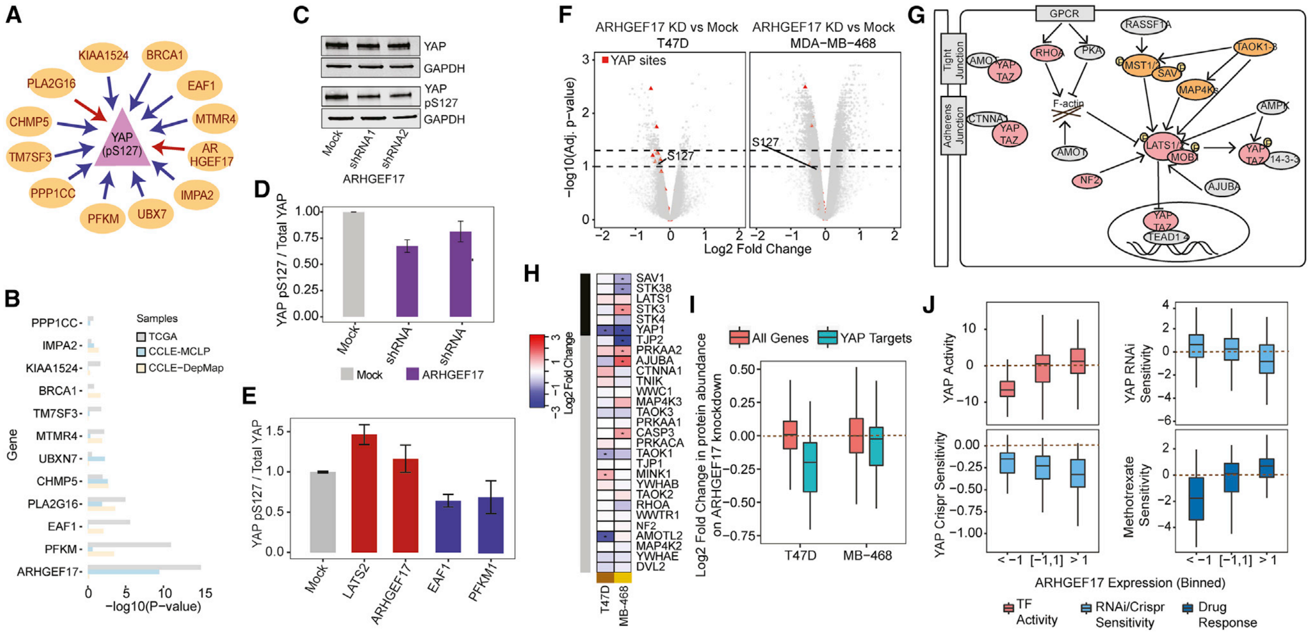

Figure 5. Experimental validation of potential regulators of YAP pS127

(A) Schematic representation of potential regulators of YAP pS127. Red and blue arrows indicate positive correlation and negative correlation, respectively.
(B) Contribution of individual regulators to YAP pS127 levels in a multiple linear regression model.
(C) Western blot analysis of YAP protein and YAP pS127 levels.
(D) Quantification of relative YAP pS127 levels on knockdown of ARHGEF17 in T47D cells.
(E) Quantification of relative YAP pS127 levels from western blot analysis on overexpression of LATS2 (positive control), ARHGEF17, EAF1, and PFKM1.
(F) Differential analysis of phosphorylation data in T47D and MDA-MB-468 cells, with YAP phosphosites highlighted in red.
(G) Schematic representation of the Hippo-signaling pathway adapted from Plouffe et al. (2016), where red and orange circles indicate critical/important components of YAP/TAZ regulation.
(H) Changes in protein abundances of components of the hippo-signaling pathway after ARHGEF17 knockdown.
(I) Comparisons of YAP cofactor activity on ARHGEF17 knockdown estimated from the proteomics data.
(J) Levels of YAP cofactor activity, RNAi/CRISPR sensitivity to loss of the YAP gene, and sensitivity to predicted drugs when binned according to ARHGEF17 levels. Error bars shown in (D) and (E) are the standard error from the mean (n = 3 independent experiments).

kinase activity has been exploited in many targeted therapies (Bhullar et al., 2018; Gross et al., 2015). We tested here the generality of such dependencies, finding them to be observed on average, although it is highly variable depending on the specific signaling protein. Our results suggest that the copy number profile of a cancer cell can, in principle, be used as a molecular fingerprint to stratify those tumors more likely to be sensitive to specific kinase inhibition. Because copy number events do not occur in isolation, additional work will be needed to understand how the combinatorial effect of multiple mutations can change the way signaling networks operate.

STAR★METHODS

Detailed methods are provided in the online version of this paper and include the following:

- KEY RESOURCES TABLE
- RESOURCE AVAILABILITY
  - Lead contact
  - Materials availability
  - Data and code availability
- EXPERIMENTAL MODEL AND SUBJECT DETAILS
- METHOD DETAILS
  - RNA interference
  - shRNA primers
  - Immunofluorescence
  - Cell lysates, immunoblotting and qRT-PCR gene expression analysis
  - Primer pairs for PCR
  - Details of (Phospho-)proteomics experiment
- QUANTIFICATION AND STATISTICAL ANALYSIS
  - Compilation of molecular and phenotypic data
  - Modeling of genetic associations
  - (Phospho-)proteomics data normalization and analysis
  - Statistical tests of experimental data

SUPPLEMENTAL INFORMATION

Supplemental information can be found online at https://doi.org/10.1016/j.celrep.2021.109155.

ACKNOWLEDGMENTS

We acknowledge Dr. Matthew Garnett (Wellcome Trust Sanger Institute), Dr. Emmanuel Goncalves (Wellcome Trust Sanger Institute), and Dr. Colm Ryan (University College Dublin) for their insightful feedback on the manuscript.

CellPress
OPEN ACCESS

D.M. is a fellow of the joint EMBL-EBI & NIHR Cambridge Biomedical Research Centre Postdoctoral (EBPOD) program. The GFP-LATS2 and GFP-ARHGEF17 clones were generously provided by Prof. Moshe Oren (Weizmann Institute of Science) and Dr. Natalia Mitin (University of North Carolina), respectively. The Fusion Lumos Orbitrap mass spectrometer was purchased with the support from a Wellcome Trust Multi-user Equipment Grant (Grant # 108467/Z/15/Z).

AUTHOR CONTRIBUTIONS

P.B. and D.M. conceived the study. P.B. and M.L.M. led the study. D.M. carried out all bioinformatics data analysis. P.B., M.L.M., D.M., and M.B.G. designed the experimental validations. M.B.G. carried out all experiments and contributed to data for the figures. C.S.D. and E.K.P. generated and processed the (phospho)proteomics dataset. D.O. contributed to the analysis. P.B. and D.M. wrote the paper, with contributions from all other authors.

DECLARATION OF INTERESTS

D.M. serves as a consultant for Curileum Discovery Ltd. All remaining authors declare no competing interests.

Received: March 19, 2019
Revised: March 2, 2021
Accepted: April 28, 2021
Published: May 18, 2021

REFERENCES

Barretina, J., Caponigro, G., Stransky, N., Venkatesan, K., Margolin, A.A., Kim, S., Wilson, C.J., Lehár, J., Kryukov, G.V., Sonkin, D., et al. (2012). The Cancer Cell Line Encyclopedia enables predictive modelling of anticancer drug sensitivity. Nature *483*, 603–607.

Bhullar, K.S., Lagarón, N.O., McGowan, E.M., Parmar, I., Jha, A., Hubbard, B.P., and Rupasinghe, H.P.V. (2018). Kinase-targeted cancer therapies: progress, challenges and future directions. Mol. Cancer *17*, 48.

Chalhoub, N., and Baker, S.J. (2009). PTEN and the PI3-kinase pathway in cancer. Annu. Rev. Pathol. *4*, 127–150.

Creixell, P., Schoof, E.M., Simpson, C.D., Longden, J., Miller, C.J., Lou, H.J., Perryman, L., Cox, T.R., Zivanovic, N., Palmeri, A., et al. (2015). Kinome-wide decoding of network-attacking mutations rewiring cancer signaling. Cell *163*, 202–217.

Curtis, C., Shah, S.P., Chin, S.-F., Turashvili, G., Rueda, O.M., Dunning, M.J., Speed, D., Lynch, A.G., Samarajiwa, S., Yuan, Y., et al.; METABRIC Group (2012). The genomic and transcriptomic architecture of 2,000 breast tumours reveals novel subgroups. Nature *486*, 346–352.

Dittmar, T., Husemann, A., Schewe, Y., Nofer, J.-R., Niggemann, B., Zänker, K.S., and Brandt, B.H. (2002). Induction of cancer cell migration by epidermal growth factor is initiated by specific phosphorylation of tyrosine 1248 of c-erbB-2 receptor via EGFR. FASEB J. *16*, 1823–1825.

Edwards, N.J., Oberti, M., Thangudu, R.R., Cai, S., McGarvey, P.B., Jacob, S., Madhavan, S., and Ketchum, K.A. (2015). The CPTAC Data Portal: A Resource for Cancer Proteomics Research. J. Proteome Res. *14*, 2707–2713.

Emdad, L., Das, S.K., Dasgupta, S., Hu, B., Sarkar, D., and Fisher, P.B. (2013). AEG-1/MTDH/LYRIC: signaling pathways, downstream genes, interacting proteins, and regulation of tumor angiogenesis. Adv. Cancer Res. *120*, 75–111.

Gao, J., Aksoy, B.A., Dogrusoz, U., Dresdner, G., Gross, B., Sumer, S.O., Sun, Y., Jacobsen, A., Sinha, R., Larsson, E., et al. (2013). Integrative analysis of complex cancer genomics and clinical profiles using the cBioPortal. Sci. Signal. *6*, pl1.

Gonçalves, E., Fragoulis, A., Garcia-Alonso, L., Cramer, T., Saez-Rodriguez, J., and Beltrao, P. (2017). Widespread Post-transcriptional Attenuation of Genomic Copy-Number Variation in Cancer. Cell Syst. *5*, 386–398.e4.

Gross, S., Rahal, R., Stransky, N., Lengauer, C., and Hoeflich, K.P. (2015). Targeting cancer with kinase inhibitors. J. Clin. Invest. *125*, 1780–1789.

Gu, J.J., Wang, Z., Reeves, R., and Magnuson, N.S. (2009). PIM1 phosphorylates and negatively regulates ASK1-mediated apoptosis. Oncogene *28*, 4261–4271.

Gumbiner, B.M., and Kim, N.-G. (2014). The Hippo-YAP signaling pathway and contact inhibition of growth. J. Cell Sci. *127*, 709–717.

Hornbeck, P.V., Kornhauser, J.M., Tkachev, S., Zhang, B., Skrzypek, E., Murray, B., Latham, V., and Sullivan, M. (2012). PhosphoSitePlus: a comprehensive resource for investigating the structure and function of experimentally determined post-translational modifications in man and mouse. Nucleic Acids Res. *40*, D261–D270.

Hyman, E., Kauraniemi, P., Hautaniemi, S., Wolf, M., Mousses, S., Rozenblum, E., Ringnér, M., Sauter, G., Monni, O., Elkahloun, A., et al. (2002). Impact of DNA amplification on gene expression patterns in breast cancer. Cancer Res. *62*, 6240–6245.

Kim, M., Kim, T., Johnson, R.L., and Lim, D.-S. (2015). Transcriptional co-repressor function of the hippo pathway transducers YAP and TAZ. Cell Rep. *11*, 270–282.

Korkut, A., Wang, W., Demir, E., Aksoy, B.A., Jing, X., Molinelli, E.J., Babur, Ö., Bemis, D.L., Onur Sumer, S., Solit, D.B., et al. (2015). Perturbation biology nominates upstream-downstream drug combinations in RAF inhibitor resistant melanoma cells. eLife *4*, e04640.

Li, J., Lu, Y., Akbani, R., Ju, Z., Roebuck, P.L., Liu, W., Yang, J.-Y., Broom, B.M., Verhaak, R.G.W., Kane, D.W., et al. (2013). TCPA: a resource for cancer functional proteomics data. Nat. Methods *10*, 1046–1047.

Li, J., Zhao, W., Akbani, R., Liu, W., Ju, Z., Ling, S., Vellano, C.P., Roebuck, P., Yu, Q., Eterovic, A.K., et al. (2017). Characterization of Human Cancer Cell Lines by Reverse-phase Protein Arrays. Cancer Cell *31*, 225–239.

Lin, J., Sampath, D., Nannini, M.A., Lee, B.B., Degtyarev, M., Oeh, J., Savage, H., Guan, Z., Hong, R., Kassees, R., et al. (2013). Targeting activated Akt with GDC-0068, a novel selective Akt inhibitor that is efficacious in multiple tumor models. Clin. Cancer Res. *19*, 1760–1772.

Macintyre, G., Goranova, T.E., De Silva, D., Ennis, D., Piskorz, A.M., Eldridge, M., Sie, D., Lewsley, L.-A., Hanif, A., Wilson, C., et al. (2018). Copy number signatures and mutational processes in ovarian carcinoma. Nat. Genet. *50*, 1262–1270.

Meng, Z., Moroishi, T., and Guan, K.-L. (2016). Mechanisms of Hippo pathway regulation. Genes Dev. *30*, 1–17.

Meyers, R.M., Bryan, J.G., McFarland, J.M., Weir, B.A., Sizemore, A.E., Xu, H., Dharia, N.V., Montgomery, P.G., Cowley, G.S., Pantel, S., et al. (2017). Computational correction of copy number effect improves specificity of CRISPR-Cas9 essentiality screens in cancer cells. Nat. Genet. *49*, 1779–1784.

Miller, M.L., Reznik, E., Gauthier, N.P., Aksoy, B.A., Korkut, A., Gao, J., Ciriello, G., Schultz, N., and Sander, C. (2015). Pan-Cancer Analysis of Mutation Hotspots in Protein Domains. Cell Syst. *1*, 197–209.

Mitin, N., Rossman, K.L., Currin, R., Anne, S., Marshall, T.W., Bear, J.E., Bautch, V.L., and Der, C.J. (2013). The RhoGEF TEM4 Regulates Endothelial Cell Migration by Suppressing Actomyosin Contractility. PLoS ONE *8*, e66260.

Moasser, M.M. (2007). The oncogene HER2: its signaling and transforming functions and its role in human cancer pathogenesis. Oncogene *26*, 6469–6487.

Moon, S., Kim, W., Kim, S., Kim, Y., Song, Y., Bilousov, O., Kim, J., Lee, T., Cha, B., Kim, M., et al. (2017). Phosphorylation by NLK inhibits YAP-14-3-3-interactions and induces its nuclear localization. EMBO Rep. *18*, 61–71.

Olow, A., Chen, Z., Niedner, R.H., Wolf, D.M., Yau, C., Pankov, A., Lee, E.P.R., Brown-Swigart, L., van 't Veer, L.J., and Coppé, J.-P. (2016). An Atlas of the Human Kinome Reveals the Mutational Landscape Underlying Dysregulated Phosphorylation Cascades in Cancer. Cancer Res. *76*, 1733–1745.

Papachristou, E.K., Kishore, K., Holding, A.N., Harvey, K., Roumeliotis, T.I., Chilamakuri, C.S.R., Omarjee, S., Chia, K.M., Swarbrick, A., Lim, E., et al. (2018). A quantitative mass spectrometry-based approach to monitor the dynamics of endogenous chromatin-associated protein complexes. Nat. Commun. *9*, 2311.

Perez-Riverol, Y., Csordas, A., Bai, J., Bernal-Llinares, M., Hewapathirana, S., Kundu, D.J., Inuganti, A., Griss, J., Mayer, G., Eisenacher, M., et al. (2019). The PRIDE database and related tools and resources in 2019: improving support for quantification data. Nucleic Acids Res. *47* (*D1*), D442–D450.

Plouffe, S.W., Meng, Z., Lin, K.C., Lin, B., Hong, A.W., Chun, J.V., and Guan, K.-L. (2016). Characterization of Hippo Pathway Components by Gene Inactivation. Mol. Cell *64*, 993–1008.

Reimand, J., and Bader, G.D. (2013). Systematic analysis of somatic mutations in phosphorylation signaling predicts novel cancer drivers. Mol. Syst. Biol. *9*, 637.

Ritchie, M.E., Phipson, B., Wu, D., Hu, Y., Law, C.W., Shi, W., and Smyth, G.K. (2015). limma powers differential expression analyses for RNA-sequencing and microarray studies. Nucleic Acids Res. *43*, e47.

Sato, S., Fujita, N., and Tsuruo, T. (2002). Interference with PDK1-Akt survival signaling pathway by UCN-01 (7-hydroxystaurosporine). Oncogene *21*, 1727–1738.

Sawyers, C. (2004). Targeted cancer therapy. Nature *432*, 294–297.

Scheid, M.P., Marignani, P.A., and Woodgett, J.R. (2002). Multiple phosphoinositide 3-kinase-dependent steps in activation of protein kinase B. Mol. Cell. Biol. *22*, 6247–6260.

Tsherniak, A., Vazquez, F., Montgomery, P.G., Weir, B.A., Kryukov, G., Cowley, G.S., Gill, S., Harrington, W.F., Pantel, S., Krill-Burger, J.M., et al. (2017). Defining a Cancer Dependency Map. Cell *170*, 564–576.e16.

Tyner, J.W., Yang, W.F., Bankhead, A., 3rd, Fan, G., Fletcher, L.B., Bryant, J., Glover, J.M., Chang, B.H., Spurgeon, S.E., Fleming, W.H., et al. (2013). Kinase pathway dependence in primary human leukemias determined by rapid inhibitor screening. Cancer Res. *73*, 285–296.

Weinstein, J.N., Collisson, E.A., Mills, G.B., Shaw, K.R., Ozenberger, B.A., Ellrott, K., Shmulevich, I., Sander, C., and Stuart, J.M.; Cancer Genome Atlas Research Network (2013). The Cancer Genome Atlas Pan-Cancer analysis project. Nat. Genet. *45*, 1113–1120.

Yang, W., Soares, J., Greninger, P., Edelman, E.J., Lightfoot, H., Forbes, S., Bindal, N., Beare, D., Smith, J.A., Thompson, I.R., et al. (2013). Genomics of Drug Sensitivity in Cancer (GDSC): a resource for therapeutic biomarker discovery in cancer cells. Nucleic Acids Res. *41*, D955–D961.

Zhao, B., Wei, X., Li, W., Udan, R.S., Yang, Q., Kim, J., Xie, J., Ikenoue, T., Yu, J., Li, L., et al. (2007). Inactivation of YAP oncoprotein by the Hippo pathway is involved in cell contact inhibition and tissue growth control. Genes Dev. *21*, 2747–2761.

Zhao, B., Li, L., Tumaneng, K., Wang, C.-Y., and Guan, K.-L. (2010). A coordinated phosphorylation by Lats and CK1 regulates YAP stability through SCF(beta-TRCP). Genes Dev. *24*, 72–85.

Zhao, W., Li, J., and Mills, G.B. (2017). Functional proteomic characterization of cancer cell lines. Oncoscience *4*, 41–42.

STAR★METHODS

KEY RESOURCES TABLE

REAGENT or RESOURCE	SOURCE	IDENTIFIER
**Antibodies**		
FLAG	ThermoFisher	Catalogue no. F1804
YAP	Cell Signaling Technology	1Catalogue no. 14074S; RRID:AB_2650491
Goat anti-Mouse IgG (H+L) Cross-Adsorbed Secondary Antibody, A lexa Fluor 488	Fisher Scientific	Catalogue no. A-11001; RRID:AB_2534069
Goat anti-Rabbit IgG (H+L) Cross-Adsorbed Secondary Antibody, Alexa Fluor 568	Fisher Scientific	Catalogue no.A-11036; RRID:AB_10563566
YAP	Cell Signaling Technology	Catalogue no. 4912S; RRID:AB_2218911
p-YAP (S127)	Cell Signaling Technology	Catalogue no. 4911S; RRID:AB_2218913
GAPDH	EMD Millipore	Catalogue no. MAB374; RRID:AB_2107445
Living Colors® A.v. Monoclonal Antibody (JL-8)	Clontech	Catalogue no. 632381; RRID:AB_2313808
**Deposited data**		
Proteomics and phosphoproteomics changes on ARHGEF17 knockdown in MDA-MB-468 and T47D Cells	PRIDE Database	PXD021031
**Experimental models: Cell lines**		
MDA-MB-468	CRUK CI/repository	N/A
T47D	CRUK CI/repository	N/A
MCF7	CRUK CI/repository	N/A
MDA-MB-361	CRUK CI/repository	N/A
HeLa	CRUK CI/repository	N/A
**Oligonucleotides**		
See Table S3 for oligonucleotide information		N/A
**Recombinant DNA**		
pZIP-SSFV-Ultra-Puro_ZsGreen	Hannon lab (CRUK)	N/A
pMDL	Hannon lab (CRUK)	N/A
pCMV-Rev	Hannon lab (CRUK)	N/A
pVSV-G	Hannon lab (CRUK)	N/A
EAF1	ORIGENE	RC207169
PFKM	ORIGENE	RC200702
GFP-LATS2	Generously provided by Prof. Moshe Oren, Weizmann Institute of Science, ISRAEL	N/A
GFP-ARHGEF17	Generously provided by Dr. Natalia Mitin, University of North Carolina, USA	N/A
**Software and algorithms**		
ImageJ		https://imagej.nih.gov/ij/
Illustrator		https://www.adobe.com
R 3.5.1		https://www.r-project.org
Bioconductor		https://www.bioconductor.org/

RESOURCE AVAILABILITY

Lead contact

Further information and requests for resources and reagents should be directed to and will be fulfilled by the lead contact, Pedro Beltrao (pbeltrao@ebi.ac.uk).

Materials availability

Plasmids generated in this study are available upon request or can be purchased from the named supplier or contact listed as supplying the reagent.

Data and code availability

The accession number for the mass spectrometry (phospho-)proteomics reported in this paper is ProteomeXchange Consortium PRIDE: PXD021031 (Perez-Riverol et al., 2019).

EXPERIMENTAL MODEL AND SUBJECT DETAILS

The human breast cancer cell lines - MDA-MB-468 (female donor), MDA-MB-361 (female donor), T47D (female donor), MCF7 (female donor) and the cervical cancer HeLa cells (female donor) were all maintained at 37°C with 5% CO2. All cells were grown in DMEM with 10% FBS. For serum starvation, cells were incubated in DMEM without other supplements for 24 h. All cell lines were authenticated using STR profiling method at the CRUK core facility.

METHOD DETAILS

RNA interference

ShRNA hairpins were designed using (http://sherwood.cshl.edu:8080/sherwood/) and cloned into the expression vector pZIP-SSFV-Ultra-Puro_ZsGreen (a generous gift of the Hannon Lab, CRUK CI). ShRNA plasmids together with pMDL, pCMV-Rev, and pVSV-G were used to produce virus in 293FT cells. Resulting viruses were used to infect and generate stable cell lines for both MDA-MB-468 and T47D using puromycin selection.

shRNA primers

ARHGEF17_shRNA1 (TGCTGTTGACAGTGAGCGCCAGGAGGTTATTCAGAGCATATAGTGAAGCCACAGATGTATATGCTCTGAATAACCTCCTGATGCCTACTGCCTCGGA)

ARHGEF17_shRNA2 (TGCTGTTGACAGTGAGCGACATCACCAAGATGGTATCTGATAGTGAAGCCACAGATGTATCAGATACCATCTTGGTGATGGTGCCTACTGCCTCGGA)

PLA2G16_shRNA1 (TGCTGTTGACAGTGAGCGCTGGAGTCATGTTCTCAAGAAATAGTGAAGCCACAGATGTATTTCTTGAGAACATGACTCCAATGCCTACTGCCTCGGA)

PLA2G16_shRNA2 (TGCTGTTGACAGTGAGCGCGAACTGCGAGCACTTTGTGAATAGTGAAGCCACAGATGTATTCACAAAGTGCTCGCAGTTCTTGCCTACTGCCTCGGA)

Immunofluorescence

HeLa cells were plated on glass cover slides in a 24 well plate at 1e5 cells/plate in DMEM-12 media with 10% FBS. 24 h later cells were transfected with the following plasmids using Lipofectamine 2000 (Thermo Fisher) in accordance with the manufacturer's instructions; GFP-LATS2 (generously provided by Prof. Moshe Oren, Weizmann Institute of Science, ISRAEL), GFP-ARHGEF17 (generously provided by Dr. Natalia Mitin, University of North Carolina, USA), Flag-tagged-EAF1 or -PFKM (purchased from GensScript). 24 h post transfection cells were fixed in 4% formaldehyde for 20 m, washed with T-PBS (0.1% Tween/PBS), and permeabilised with 0.1% Triton X-100 for 15 m. After blocking for 1h (blocking buffer, 3% BSA in T-PBS), fixed cells were incubated with primary antibody to FLAG (F1804/ThermoFisher) and YAP (14074S/Cell Signaling Technology) in blocking buffer for 1h. Following extensive washing with T-BPS slides were incubated with Alexa conjugated antibodies for FLAG and YAP detection and then mounted with Prolong Antifade mounting. Images were captured in a Leica SP5 confocal microscope and processed using Fiji software.

Cell lysates, immunoblotting and qRT-PCR gene expression analysis

For GFP-TEM4/GFP-LATS2 overexpression studies, cells were seeded at low density (50% confluency), in contrast to EAF1/PFKM overexpression studies were cells were seeded at high density (100% confluency). Both transfected and shRNA KD cells were lysed in 50 mM Tris-Cl, pH 7.6; 150 mM NaCl, 1% NP-40 supplemented with Protease inhibitors (cOmplete EDTA free and PhosStop phosphatase inhibitor, Sigma). Cells lysates were centrifuged for 10 min at 4oC to remove insoluble debris. Lysates were separated using 4%–20% Gradient gels (Miniprotein TGXTM Gel, Biorad) and immunoblotted using standard protocol. Primary antibodies used were YAP (4912S/Cell Signaling Technology, 1:1000), p-YAP (S127)(4911S/ Cell Signaling Technology, 1:1000) and GAPDH (MAB374/

EMD Millipore, 1:5000). Blots were probed with mouse or rabbit specific IRDye 800 (LiCOR) and acquired, and quantified, using Odyssey Classic. Total RNA was isolated and purified from cells using miRNeasy Mini Kit (QIAGEN). cDNA was synthesized using the High Capacity RNA-to-cDNA kit (ABI) according to manufacturer's instructions. qRT-PCR was performed using Fast SYBR Green Master Mix (ABI) on the QuantStudio 12K Flex Real-Time PCR System (ABI). Relative expression levels were defined using the DDCt method and normalizing to 36B4/RPLP0.

Primer pairs for PCR

PLA2G16: primer pair 1 (AGGCCATCGTGAAGAAGGAA & CAAAGTGCTCGCAGTTCTCA), primer pair 2 (AGGAGGTGCTCTACAAGCTG & CTCCATAGCGCAGCTCATTC). ARHGEF17: primer pair 1 (GAGAAGTTGAGCCCATGCTG & GTAGGGCCCTTTCAGACTGT), primer pair 2 (GCGGAAGTCCCTGTCAAATC & ACCCTCAGCTCTGAAAGGTC). 36B4/RPLP0: primer pair (GTGTTCGACAATGGCAGCAT & GACACCCTCCAGGAAGCGA)

Details of (Phospho-)proteomics experiment

Adherent cells were grown to complete confluence prior to harvesting and harvested by scraping on ice in 1X PBS containing protease inhibitors (PhosSTOP^{TM} and cOmplete Protease Inhibitor Cocktail, Merck). Cell pellets were dissolved as described previously (Papachristou et al., 2018) and proteins were trypsin-digested (Pierce) and labeled using TMT labels (Thermo Scientific, TMT11plex reagents). The TMT peptide mixture was fractionated on a Dionex Ultimate 3000 system at high pH using a C18 column (3.5 μm 2.1x150mm, Xbridge Waters). Each fraction was split for full proteome analysis (10%) and phosphoproteome analysis (90%). The fractions for the phospho-proteome analysis were enriched using the High-Select Fe-NTA Phosphopeptide Enrichment kit (Thermo Scientific, #A32992) according to manufacturer's instructions. The Phosphopeptide-enriched fractions were analyzed on the Q-Exactive HF (Thermo Scientific) coupled with the Dionex Ultimate 3000 UHPLC system. For the peptide separation the EASY-spray analytical column 75 μm × 25cm, C18, 2 μm, 100 Ȧ column was used for multi-step gradient elution. The full scan was performed in the Orbitrap in the range of 400-1,600 m/z at 60k resolution. For MS2, the 10 most intense fragments were selected at resolution 30K. A 2.0Th isolation window was used and the HCD collision energy was set up at 33%. The total proteome analysis was performed with the nano-ESI Fusion-Lumos (Thermo Scientific) system. The full scans were performed in the Orbitrap in the range of 380-1,500 m/z at 120K resolution and the MS2 scans were performed in the ion trap with collision energy 35%. Peptides were isolated in the quadrupole with isolation window 0.7Th. The 10 most intense fragments were selected for Synchronous Precursor Selection (SPS) HCD-MS3 analysis with MS2 isolation window 2.0 and HCD collision energy 55%. The detection was performed with Orbitrap resolution 50k and in scan range 100-400 m/z. Both total proteome and phospho-proteome raw files were processed with the SequestHT search engine on Proteome Discoverer 2.1 software for peptide and protein identification. The data were searched against a uniprot database containing human reviewed entries. The SequestHT node included the following parameters: Precursor Mass Tolerance 20 ppm, Fragment Mass Tolerance 0.5 for the CID spectra and 0.02 for the HCD spectra, Dynamic Modifications were Oxidation of M (+15.995 Da), Deamidation of N, Q (+0.984 Da) and Static Modifications were TMT6plex at any N terminus/K (+229.163 Da) and Methylthio at C (+45.988). Phospho at S, T, Y (+79.966) was included for the phospho-proteome and the ptmRS node was used for the confidence of the localization of the phospho-sites. Only unique peptides filtered for FDR $< 1\%$ were used for quantification and the consensus workflow included calculation of TMT signal-to-noise.

QUANTIFICATION AND STATISTICAL ANALYSIS

Compilation of molecular and phenotypic data

Normalized copy number (log2) datasets (10,654 samples) and RNaseq expression datasets (9,548 samples) for 31 different tumor types generated as part of TCGA (Weinstein et al., 2013) consortium were obtained from cBioPortal (Gao et al., 2013) database. Normalized copy number (log2) dataset (995 cell lines) and RNaseq expression dataset (967 cell lines) were obtained from cancer cell lines (CCLE) (Barretina et al., 2012). Normalized RPPA (phospho)proteomics datasets for 31 different tumor types consisting of 7694 samples were obtained from TCPA (Li et al., 2013) database. Normalized RPPA (phospho)proteomics dataset were downloaded for 651 cancer cell lines from MCLP (v1.1)(Li et al., 2017) and 899 cancer cell lines from DepMap.

Genome-wide RNAi (Tsherniak et al., 2017) screen and CRISPR (Meyers et al., 2017) screens were obtained from the Broad Institute portal (https://depmap.org/portal/achilles/). These datasets were generated by Project Achilles to catalog gene essentiality across cancer cell lines. The RNAi screen (Ach 2.20.2) has high coverage targeting 17,098 genes in 501 cell lines. Out of these, 406 cell lines had DepMap-RPPA data and 166 cell lines had available MCLP-RPPA data (Li et al., 2017) permitting pan-cancer correlative analysis. The CRISPR screen (Avana CRISPR-Cas9) targets 17,670 genes across 517 cell lines. 420 of these cell lines also had DepMap-RPPA data and 163 cell lines also had MCLP-RPPA data. IC_{50} values of sensitivity to inhibitors/drugs were obtained from Genomics of Drug Sensitivity in Cancer Database (Yang et al., 2013). The dataset comprises sensitivity values for 265 drugs across 746 cell lines. These cell lines were mapped with those in CCLE to identify 596 cell lines with DepMap-RPPA estimates and 259 cell lines with MCLP-RPPA estimates.

Modeling of genetic associations

A total of 6,558 samples were compiled with information on Copy Number, Expression and phosphorylation levels measured with RPPA for patient samples from TCGA. We restricted our analysis to 5598 samples from 17 tumor types which had more than 150 samples. We predicted associations between 18,350 CNVs and 37 RPPA phosphosites across 17 different tumor types using linear mixed effect models. Each linear model had phosphosite levels as response variable, CNV as the explanatory variable and matched protein abundance (same as phosphosite) and tumor type as covariates. A separate covariate model was run for each phosphosite.

$$\text{Phosphosite} \sim \text{CNV Model}: \; Psx = Cy + Prx + Tt$$

$$\text{Covariate Model}: \; Psx = Prx + Tt$$

Where *Psx* and *Prx* are the RPPA phosphosite and protein abundance measurements respectively of site *x, Cy* is the CNV measurements for tumor samples of gene *y* and *Tt* is the tissue type of the tumor samples.

Two models were compared using a likelihood ratio test to identify the effect of a given CNV on phosphosite levels after taking into account the matched protein abundance and tumor specificity. In order to identify associations between gene expression and phosphosite levels, the same approach was repeated after replacing the copy number measurements (*Cy*) with expression-based measurements (*Ey*) in the model.

A network-based score to rank genes was derived by calculating a network-based score between the CNV gene and phosphosite gene in the STRING network.

$$\text{Network Score} = \sum W \Big/ L^2$$

Where *W* and *L* are edge weights and length of the shortest path between the CNV gene and the phosphosite gene in the STRING network respectively.

Genes that were found to be significant at both CNV and Expression levels with each phosphosite were short-listed. In order to prioritise a 'causal' gene within a copy number segment, we ranked the genes based on the combined rank of i) effect size of CNV~Phosphosite levels, ii) effect size of Expression~Phosphosite levels and iii) the network based score. Short-listed genes for a phosphosite were ordered by their highest average combined rank, calculated using Borda method in R. Genes were iteratively picked with priority given to the highest ranked gene over other genes with strongly correlated copy number profile ($r > 0.5$), which made them more likely to belong to the same genomic segment.

Significant associations identified in tumor samples were also tested in 890 cancer cell lines with RPPA and expression data in DepMap database and 319 cancer cell lines with Expression and RPPA datasets. To evaluate the quality of our associations from the pan-tumor analysis, we predicted the phosphosite levels from expression data in cancer cell lines using models trained using TCGA, MCLP and DepMap data and correlated with the 'true' phosphosite activities measured independently in MCLP or DepMap databases.

Similar to genetic associations performed between gene copy number and phosphosite levels, associations were also performed between drug screens and phosphosite levels across cancer cell lines using a linear model approach. Briefly, drug response data (Log IC_{50} values) generated from drug treatment of cancer cell lines were associated with levels of individual phosphosites across these cancer cell lines with the protein abundance of the phosphosite as a covariate in the linear model. The *p-value*s of these drug~gene associations were adjusted for multiple comparisons and used as a measure of significance.

(Phospho-)proteomics data normalization and analysis

Quantification of 65,535 peptides were generated for both T47D and MDA-MB-468 cells. The data were quantile normalized separately for each cell line and summarized at protein level based on the median abundance of all the peptides mapped to each protein in a sample. Therefore, a total of protein abundance measurements were obtained for 7,963 and 7,943 proteins from T47D and MDA-MB-468 cells respectively with 7,119 proteins common between the two cell lines. Differential proteome analysis between siARHGEF17 and matched siRNA control samples was performed separately for T47D cell line and MDA-MB-468 cell line using the lmFit and the eBayes functions in the limma (Ritchie et al., 2015) package in R. In the proteome analysis, significantly changing proteins with 5% FDR cutoff were identified. Downstream targets of YAP transcription co-factor were obtained from Kim et al. (2015) from which we retained only positively regulated targets for our analysis.

The phosphoproteomics measurements were obtained for 41,249 (27,601 unique peptides) providing quantification for 34,716 unique phosphosites in T47D cell line. Similarly, there were 40,754 phosphopeptides (27,815 unique peptides) providing quantification for 34,223 unique phosphosites in MDA-MB-468 cell line. The data were quantile normalized for each cell line separately. Differential phosphoproteome analysis between siARHGEF17 and matched siRNA control samples was performed separately for T47D cell line and MDA-MB-468 cell line using the lmFit and the eBayes functions in the limma (Ritchie et al., 2015) package in R. In the phosphoproteomics analysis, significantly changing phosphopeptides with 5% FDR cutoff were identified.

Statistical tests of experimental data

All statistical tests were performed in R. The two-tailed paired t test was applied to analyze differences between control and the positive (or negative) regulators in the knockdown and overexpression experiments. Data are shown as mean + SEM relative to control experiment.

Cell Reports, Volume 35

Supplemental information

Copy number aberrations drive kinase rewiring, leading to genetic vulnerabilities in cancer

Danish Memon, Michael B. Gill, Evangelia K. Papachristou, David Ochoa, Clive S. D'Santos, Martin L. Miller, and Pedro Beltrao

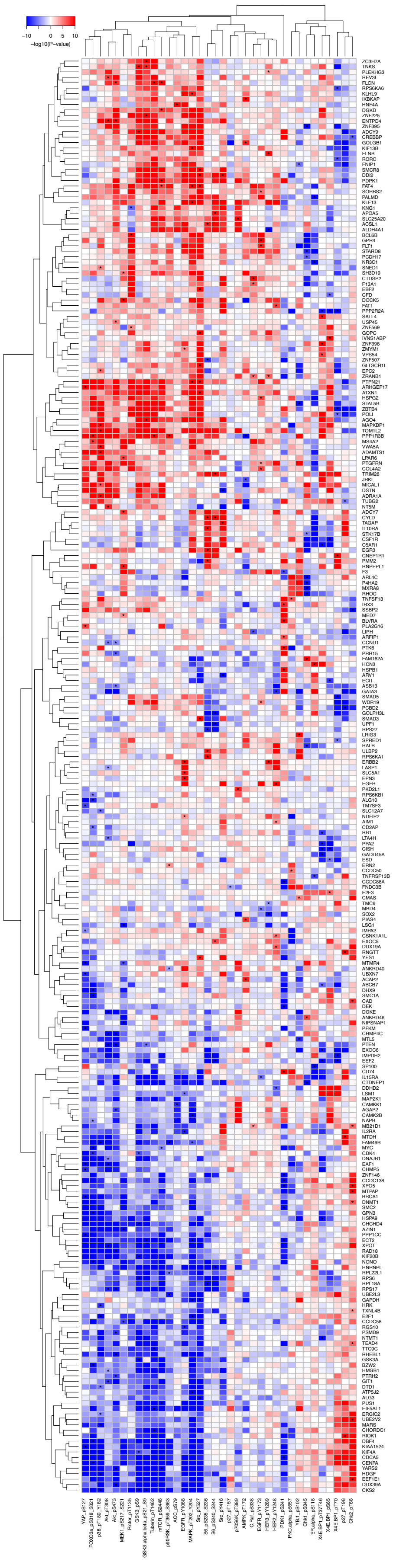

Figure S1. Association between the expression of the predicted driver genes and the activities of the RPPA phosphosites in the TCGA cohort. Significant associations identified from the CNV-phosphosite pipeline are indicated with '*'. Related to Figures 1 and 2.

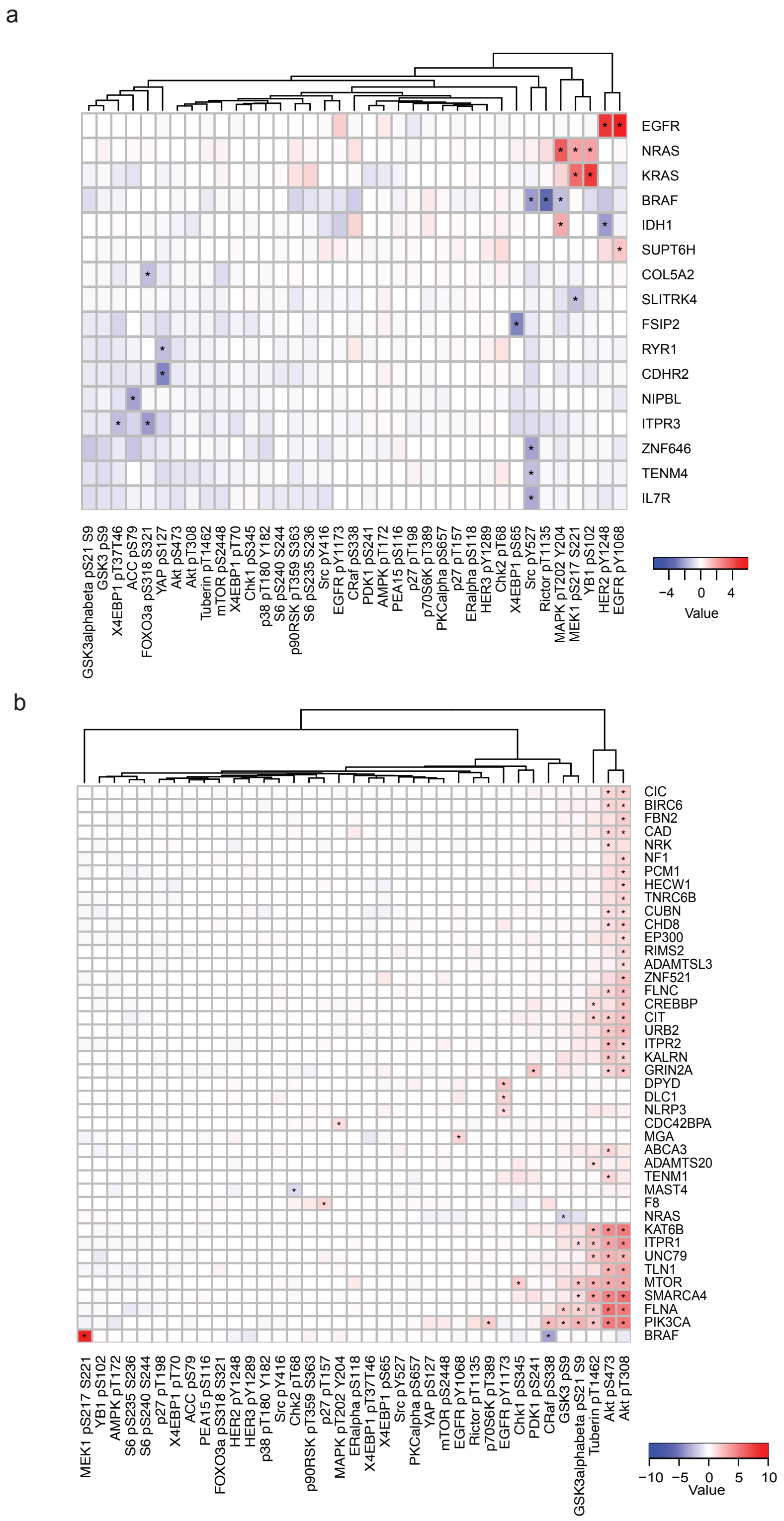

Figure S2. Association between recurrent missense mutations in genes and phosphosite activities in the TCGA cohort and CCLE cohort. (a) The TCGA analysis was restricted to genes with missense mutations in more than 50 out of 4,150 TCGA tumour samples. Only genes with significant association with atleast one phosphosite are shown. * indicates significant associations (FDR < 5%). (b) The CCLE analysis was restricted to genes with missense mutations in more than 50 out of 905 cancer cell lines. Only genes with significant association with atleast one phosphosite are shown. * indicates significant associations (FDR < 5%). Related to Figure 2.

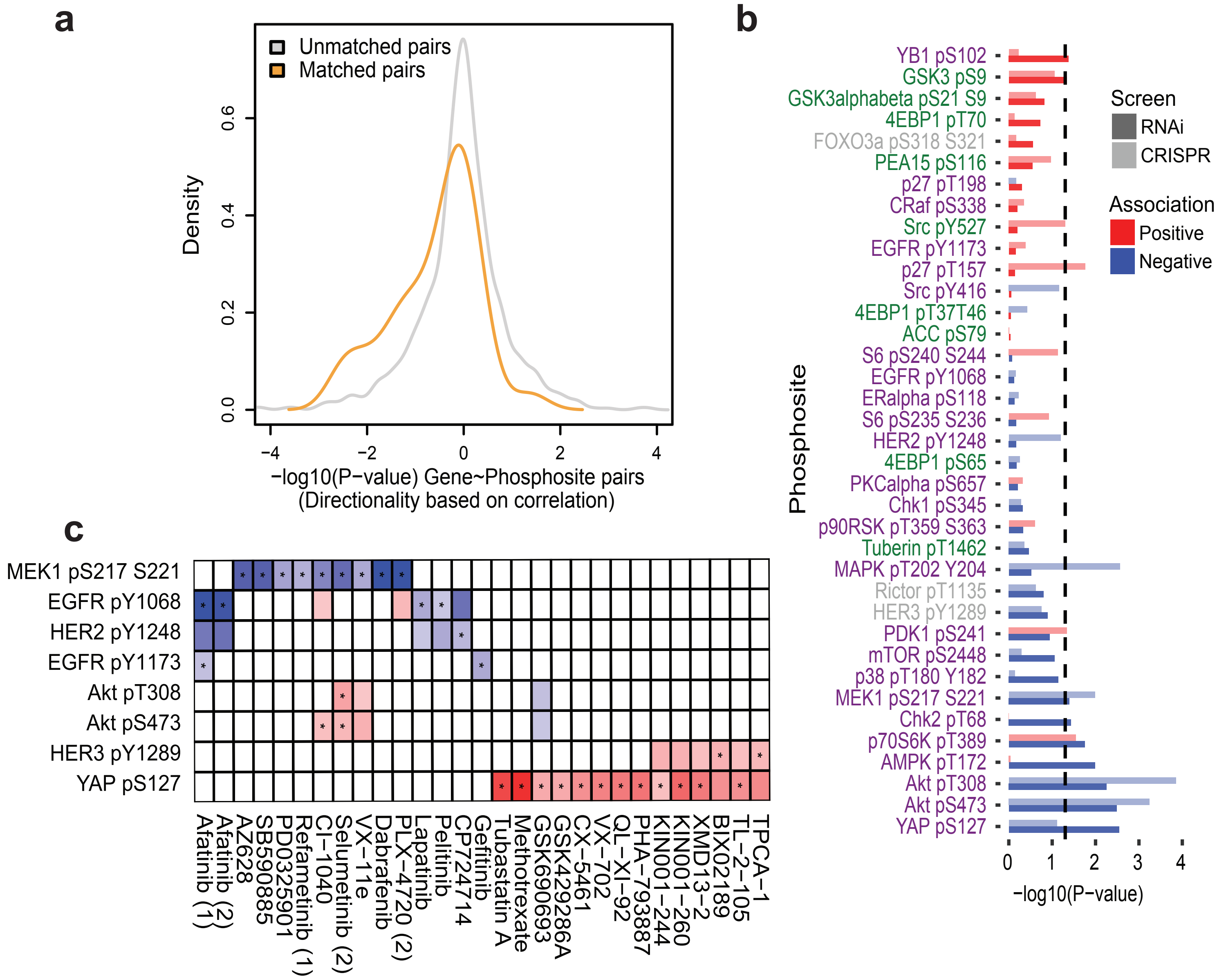

Figure S3. Evidence of kinase addiction in MCLP cancer cell lines. (a) Distribution for the significance of association (-log10(p-value)) between phosphosite activity and gene essentiality (RNAi). (b) significance of association (-log10(p-value)) between phosphosite activity and gene essentiality for each phosphosite using both RNAi and CRISPR Screen. (c) Association between phosphosite activity and drug sensitivity (1% FDR). Positive and negative associations and also supported by RNAi~Drug response are indicated in red and blue respectively. * indicates associations also supported by CRISPR~Drug association. Related to Figure 3.

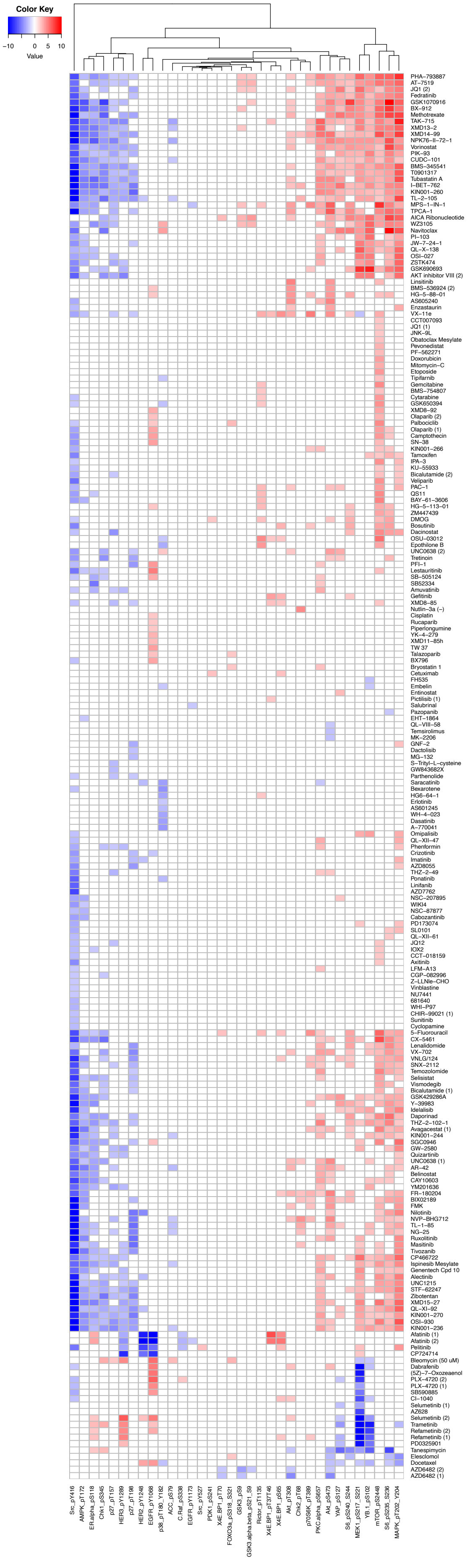

Figure S4. Association between phosphosite activity and drug sensitivity in cancer cell lines. Positive and negative associations that were found to be significant are indicated in red and blue respectively. Related to Figure 3.

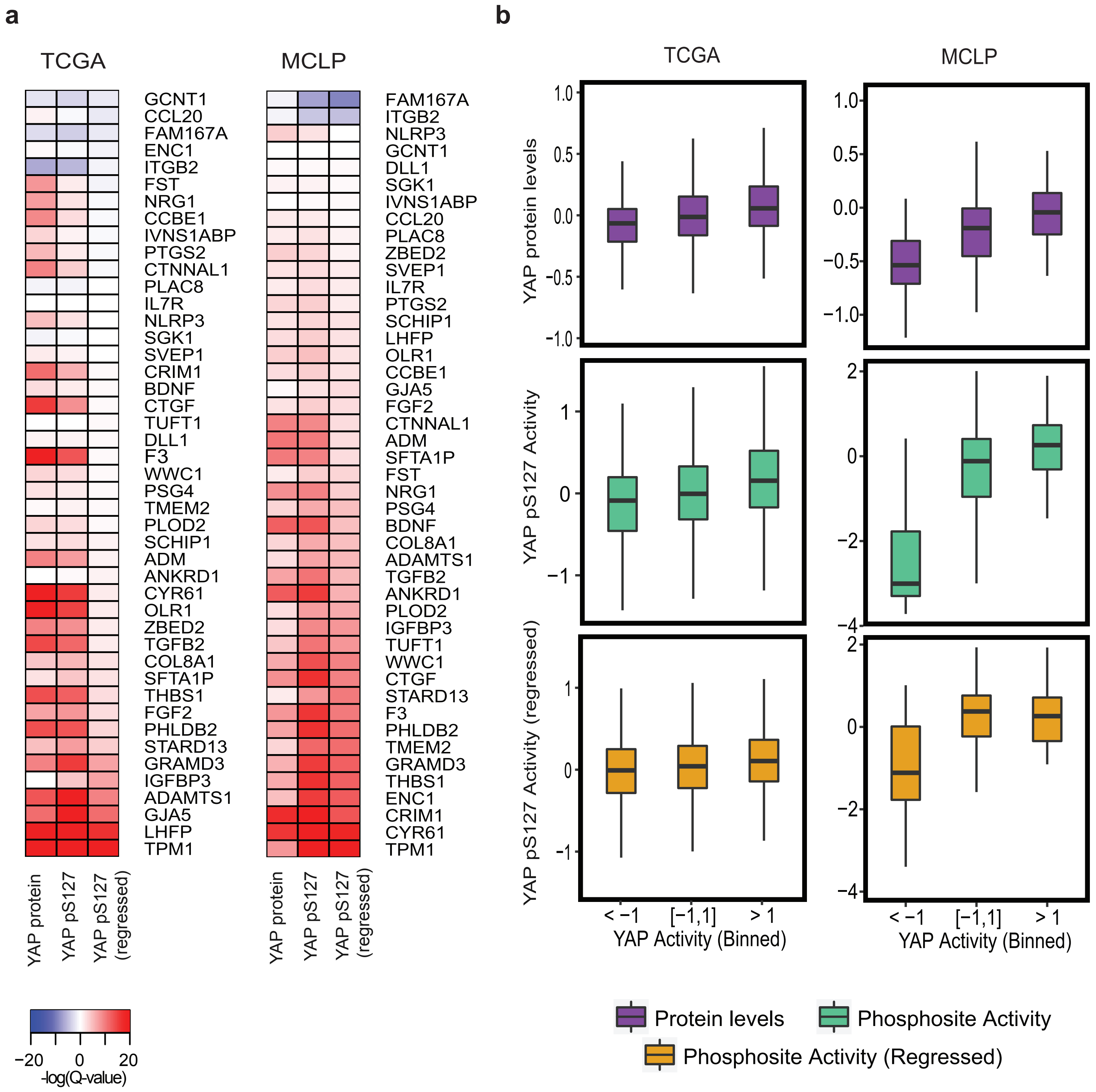

Figure S5. Relationship between YAP protein and YAP pS127 activity and downstream targets of YAP co-factor in TCGA and MCLP Dataset. (a) Correlation between YAP targets and YAP protein and YAP pS127 activity. (b) Difference in YAP protein, YAP and phosphosite levels (before and after regressing out protein changes), binned by levels of YAP co-fac-tor activity. Related to Figure 5.

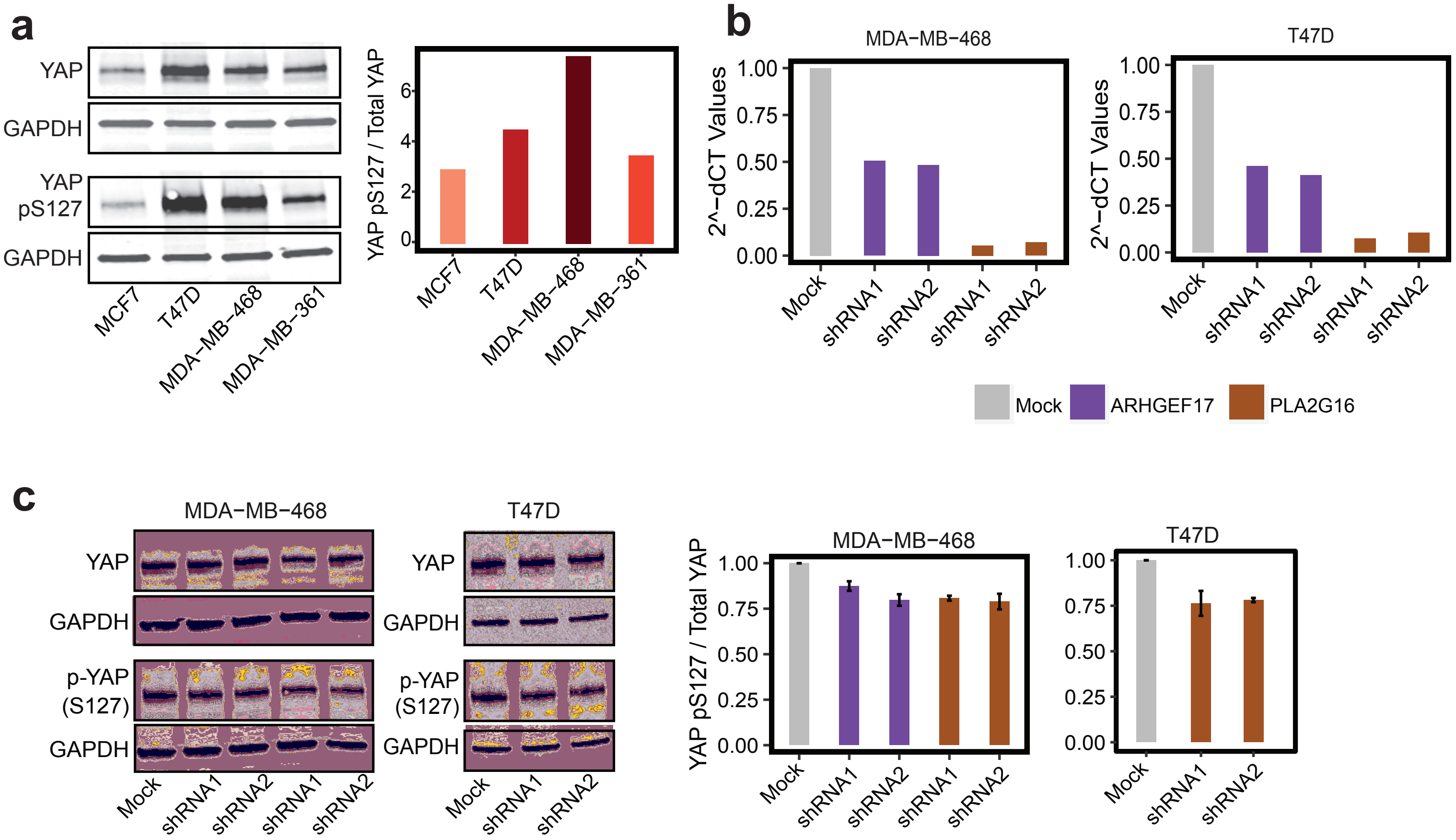

Figure S6 - Knockdown of ARHGEF17 and PLA2G16 modulates the levels of YAP pS127. (a) Western blot analysis of YAP protein and YAP pS127 levels and quantification of relative levels of YAP pS27 in MCF7, T47D MDA-MB-468 and MDA-MB-361 cells. (b) qRT-PCR of ARHGEF17 and PLA2G16 expression levels on shRNA mediated knockdown of ARHGEF17 and PLA2G16 in T47D and MDA-MB-468 cells. (c) Western blot analysis of YAP protein and YAP pS127 levels and quantification of relative YAP pS127 levels on knockdown of ARHGEF17 and PLA2G16 in T47D and MDA-MB-468 cells. Related to Figure 5.

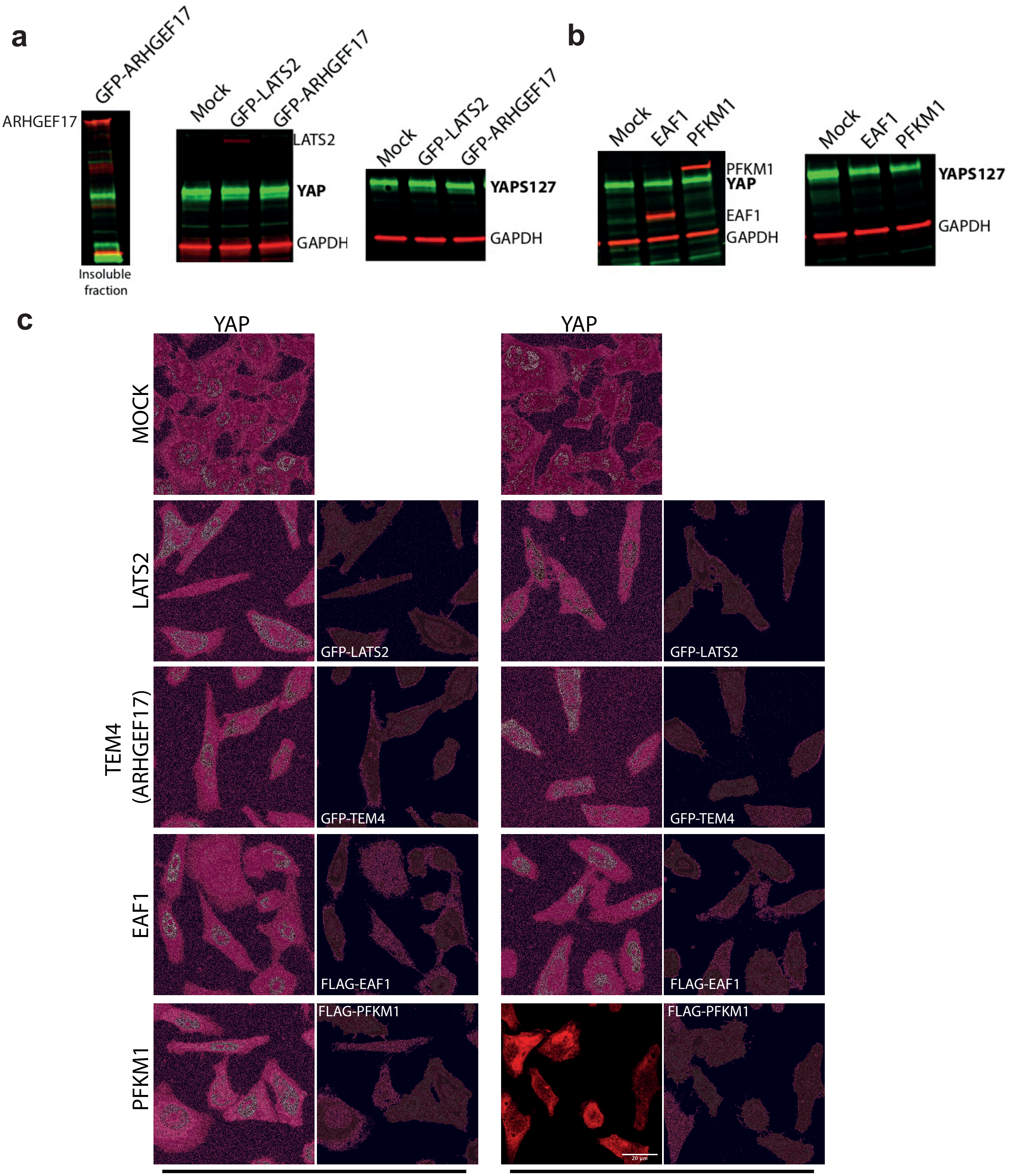

Figure S7 - Over-expression of predicted regulators of hippo-signalling modulates the levels of YAP pS127 and regulates YAP cellular localization. (a) Western blot analysis of YAP protein and YAP pS127 levels on over-expression of ARHGEF17 and LATS2 (positive control). (b) Western blot analysis of YAP protein and YAP pS127 levels on over-expression of EAF1 and PFKM1. (c) Immunofluorescence of YAP protein and YAP pS127 in HeLa cells overexpressing MOCK (control), GFP-LATS2 (positive control), GFP-ARHGEF17, GFP-EAF1 and GFP-PFKM1. Related to Figure 5.

Oligonucleotide	Source
ARHGEF17_shRNA1 (TGCTGTTGACAGTGAGCGCCAGGAGGTTATTCAGAGCATATAGTGAAGCCACAGATGTATATGCTCTGAATAACCTCCTGATGCCTACTGCCTCGGA)	Sigma
ARHGEF17_shRNA2 (TGCTGTTGACAGTGAGCGACATCACCAAGATGGTATCTGATAGTGAAGCCACAGATGTATCAGATACCATCTTGGTGATGGTGCCTACTGCCTCGGA)	Sigma
PLA2G16_shRNA1 (TGCTGTTGACAGTGAGCGCTGGAGTCATGTTCTCAAGAAATAGTGAAGCCACAGATGTATTTCTTGAGAACATGACTCCAATGCCTACTGCCTCGGA)	Sigma
PLA2G16_shRNA2 (TGCTGTTGACAGTGAGCGCGAACTGCGAGCACTTTGTGAATAGTGAAGCCACAGATGTATTCACAAAGTGCTCGCAGTTCTTGCCTACTGCCTCGGA)	Sigma
Primer pairs for PCR:<br>PLA2G16: primer pair 1 **(**AGGCCATCGTGAAGAAGGAA & CAAAGTGCTCGCAGTTCTCA)	Sigma
Primer pairs for PLA2G16; PCR:primer pair 2 **(**AGGAGGTGCTCTACAAGCTG & CTCCATAGCGCAGCTCATTC).	Sigma
Primer pairs for PCR:<br>ARHGEF17**:** primer pair 1 **(**GAGAAGTTGAGCCCATGCTG & GTAGGGCCCTTTCAGACTGT),	Sigma
Primer pairs for PCR:<br>ARHGEF17**:** primer pair 2, primer pair 2 **(**GCGGAAGTCCCTGTCAAATC & ACCCTCAGCTCTGAAAGGTC)**.**	Sigma
36B4/RPLP0: primer pair (GTGTTCGACAATGGCAGCAT & GACACCCTCCAGGAAGCGA)	Sigma

Table S1 - Oligonucleotide information. Related to STAR Methods.
